# Lower Microscopy Sensitivity with Decreasing Malaria Prevalence in the Urban Amazon Region, Brazil, 2018–2021

**DOI:** 10.3201/eid3009.240378

**Published:** 2024-09

**Authors:** Priscila T. Rodrigues, Igor C. Johansen, Winni A. Ladeia, Fabiana D. Esquivel, Rodrigo M. Corder, Juliana Tonini, Priscila R. Calil, Anderson R.J. Fernandes, Pablo S. Fontoura, Carlos E. Cavasini, Joseph M. Vinetz, Marcia C. Castro, Marcelo U. Ferreira, on behalf of the Mâncio Lima Cohort Study Working Group

**Affiliations:** Centro Nacional de Pesquisa em Energia e Materiais, Campinas, Brazil (P.T. Rodrigues);; University of São Paulo, São Paulo, Brazil (P.T. Rodrigues, I.C. Johansen, W.A. Ladeia, F.D. Esquivel, R.M. Corder; J. Tonini, P.R. Calil, A.R.J. Fernandes, P.S. Fontoura, M.U. Ferreira);; State University of Campinas, Campinas (I.C. Johansen);; Universidade Federal do ABC, Santo André, Brazil (J. Tonini);; Instituto Federal de Educação, Ciência e Tecnologia de Minas Gerais, Belo Horizonte, Brazil (A.R.J. Fernandes);; Ministry of Health, Brasília, Brazil (P.S. Fontoura);; Faculdade de Medicina de São José do Rio Preto, São José do Rio Preto, Brazil (C.E. Cavasini);; Yale School of Medicine, New Haven, Connecticut, USA (J.M. Vinetz);; Universidad Peruana Cayetano Heredia, Lima, Peru (J.M. Vinetz);; Harvard T.H. Chan School of Public Health, Boston, Massachusetts, USA (M.C. Castro);; Global Health and Tropical Medicine, NOVA University of Lisbon, Lisbon, Portugal (M.U. Ferreira);; Institute of Hygiene and Tropical Medicine, NOVA University of Lisbon (M.U. Ferreira)

**Keywords:** malaria, Amazon Basin, Plasmodium vivax, Plasmodium falciparum, epidemiology, microscopy, urban, vector-borne infections, Brazil, South America, parasites

## Abstract

Malaria is increasingly diagnosed in urban centers across the Amazon Basin. In this study, we combined repeated prevalence surveys over a 4-year period of a household-based random sample of 2,774 persons with parasite genotyping to investigate the epidemiology of malaria in Mâncio Lima, the main urban transmission hotspot in Amazonian Brazil. We found that most malarial infections were asymptomatic and undetected by point-of-care microscopy. Our findings indicate that as malaria transmission decreases, the detection threshold of microscopy rises, resulting in more missed infections despite similar parasite densities estimated by molecular methods. We identified genetically highly diverse populations of *Plasmodium vivax* and *P. falciparum* in the region; occasional shared lineages between urban and rural residents suggest cross-boundary propagation. The prevalence of low-density and asymptomatic infections poses a significant challenge for routine surveillance and the effectiveness of malaria control and elimination strategies in urbanized areas with readily accessible laboratory facilities.

Despite recent progress toward elimination, persisting malaria transmission in the Americas continues to pose a risk for infection to 120 million persons (*1*). The Amazon Basin, spanning 9 countries of South America, accounts for ≈90% of the 600,000 laboratory-confirmed cases recorded annually on the continent (*2*). The actual malaria burden may be significantly underestimated because point-of-care microscopy is not sensitive enough to detect the parasites in all low-density infections, most of which are asymptomatic, in the Amazon Basin (*3*–*5*).

Traditionally considered a rural disease, malaria is now emerging in rapidly growing urban centers across the Amazon Basin (*6*–*8*). After decades of massive rural-to-urban migration, 75% of the population in the Brazilian Amazon now resides in settlements classified as urban according to municipality law (https://censo2022.ibge.gov.br/panorama). This classification is adopted by the Brazilian Institute of Geography and Statistics, the country’s census bureau. Urban health facilities, generally more accessible and better equipped than their rural counterparts, can provide prompt malaria diagnosis and treatment and prevent new infections (*9*). However, chronic asymptomatic and submicroscopic infections can escape detection and sustain transmission. The rapid, unplanned urbanization of this region may transform malaria into an urban disease in the Amazon Basin, with broad public health implications.

In this study, we combined population-based prevalence data and parasite genotyping to examine the epidemiology of residual malaria in the main urban transmission hotspot of Brazil. We tested the hypothesis that, once introduced into receptive urbanized spaces, malaria parasites spread unnoticed among asymptomatic carriers, potentially causing outbreaks more frequently than parasite lineages restricted to remote rural villages.

The Institutional Review Board of the Institute of Biomedical Sciences, University of São Paulo, and the National Committee on Ethics in Research of the Ministry of Health of Brazil approved this study protocol (CAAE no. 6467416.6.0000.5467). We obtained written informed consent and assent from all study participants or their parents or guardians.

## Methods

### Study Site

**Figure 1:**
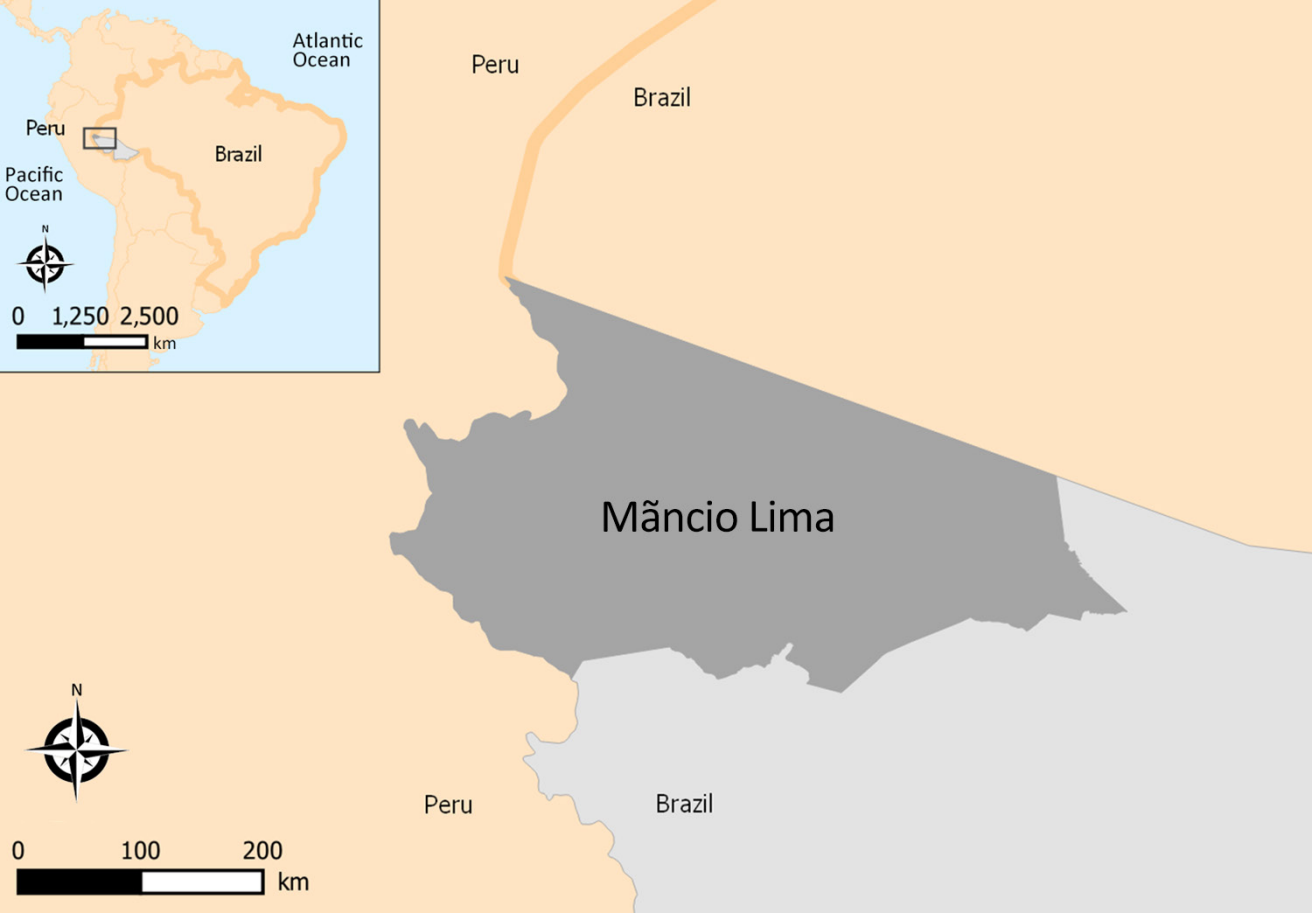
Location of the municipality of Mâncio Lima, Brazil, the study site for study of microscopy sensitivity and decreased malaria prevalence in the urban Amazon Region, Brazil, 2018–2021. Mâncio Lima (dark gray shading), population 19,294 in 2022, is situated in the upper Juruá Valley region (light gray shading) of the western Brazilian Amazon, adjacent to the border with Peru, covering an area of 5,453 km^2^. Insert map shows location of study area in South America.

The municipality or county of Mâncio Lima (Figure 1) (*7*) had an annual parasite incidence (number of laboratory-confirmed malaria cases per 1,000 persons per year) estimated at 422.8, the highest in Brazil, at the study onset in 2018. Half of the municipality’s population lived in the town of Mâncio Lima (Appendix Figure 1), which accounted for 48% of the municipality’s malaria cases in 2018. *Anopheles* (*Nyssorhynchus*) *darlingi,* the primary malaria vector, thrives in fish farming tanks and ponds that were opened across the town starting in the early 2000s (*10*).

### Household Panel Survey

**Figure 2:**
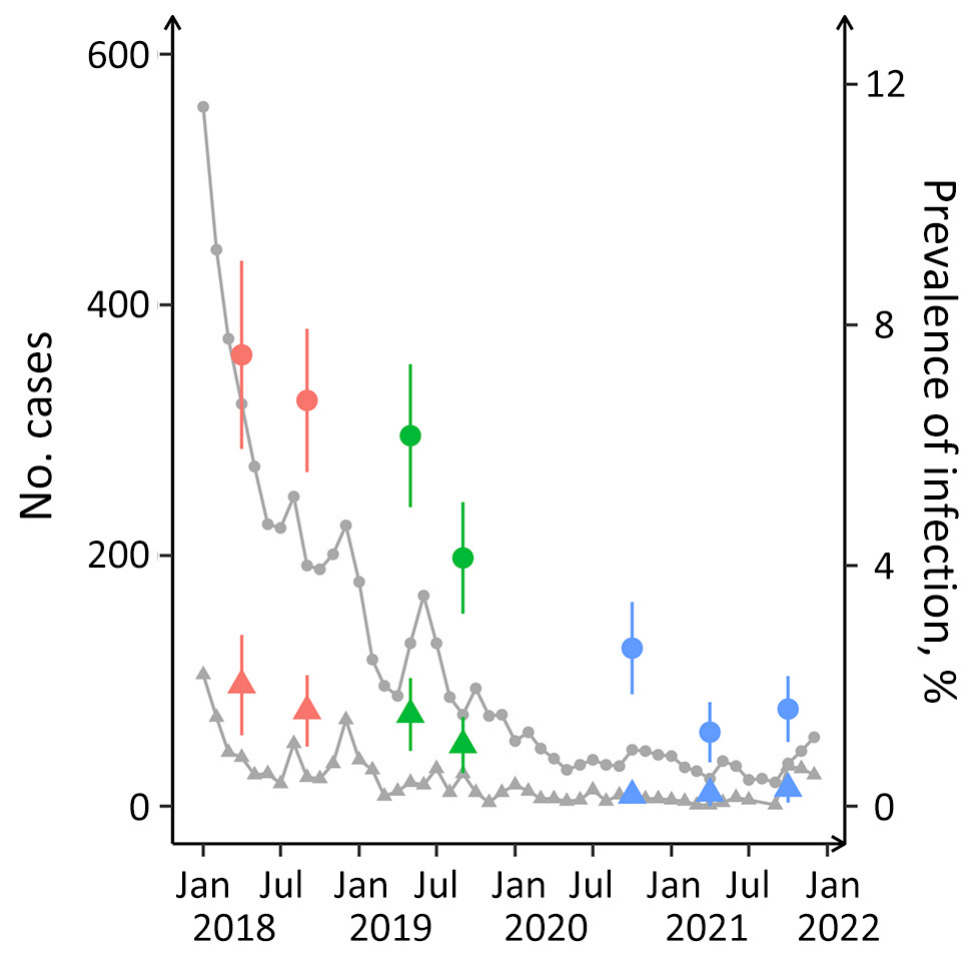
Monthly counts of microscopy-confirmed cases and percentages of infections diagnosed by PCR for *Plasmodium vivax* and *P. falciparum* in study of microscopy sensitivity and decreased malaria prevalence in the urban Amazon Region, Brazil, January 2018–December 2021. Circles indicate *Plasmodium vivax* and triangles *P. falciparum.* Red circles and triangles represent study waves 1 (April‒May 2018) and 2 (September‒October 2018); green, waves 3 (May‒June 2019) and 4 (September‒October 2019); blue, waves 5 (October‒November 2020), 6 (April‒May 2021), and 7 (October‒November 2021). Error bars indicate 95% CIs for prevalence rates. Anonymized malaria case notifications were downloaded from the electronic database of the Ministry of Health of Brazil (http://200.214.130.44/sivep_malaria)

We analyzed biological specimens and data from a household-based random sample comprising 20% of the residents in the town of Mâncio Lima (*11*). Our analysis included repeated measurements on a cohort of residents in the same households to characterize temporal changes in the outcomes of interest (*12*). Study waves took place in April‒May 2018 (wave 1), September‒October 2018 (wave 2), May‒June 2019 (wave 3), September‒October 2019 (wave 4), October‒November 2020 (wave 5), April‒May 2021 (wave 6), and October‒November 2021 (wave 7). Over the 4-year study period, the incidence of microscopy-confirmed malaria decreased markedly among urban residents (Figure 2).

We recorded sociodemographic and illness information and used a household-based wealth index as a proxy of socioeconomic status (Appendix). We collected finger-prick capillary blood samples from participants >3 months of age, regardless of any symptoms, for malaria diagnosis and parasite genotyping. We asked participants whether they had experienced any signs or symptoms that might have been caused by a malarial infection <7 days before the interview. We specifically asked those reporting any signs or symptoms whether they had fever, chills, or headache.

### Laboratory Diagnosis of Malaria

We stained thick blood smears with Giemsa (Merck KGaA, https://www.emdgroup.com) and examined for malaria parasites under 1,000× magnification; a research microscopist who had >15 years of experience reviewed >200 fields. The protocols for slide staining and reading remained consistent throughout the study period. We also tested blood samples for malarial antigens, during waves 3 and 4, using the QuickProfile Pf/Pv (Lumiquick, https://lumiquick.com) rapid diagnostic test, and used them for DNA extraction followed by molecular diagnosis, during all study waves (Appendix).

We initially screened samples with a genus-specific PCR that targets a conserved sequence of the *cytb* gene of human-infecting malaria parasites (*13*) using a detection threshold of 0.2 amplicon copies/µL, equivalent to as few as 4 parasites/mL, assuming an average of 50 mitochondrial genome copies per blood-stage parasite. We further tested positive samples with separate species-specific quantitative TaqMan assays (ThermoFisher Scientific, https://www.thermofisher.com) that targeted mitochondrial genome sequences of *P. vivax* (84-bp domain of the *cox1* gene) or *P. falciparum* (90-bp domain spanning the 3′ end of the *cox1* gene and the nearby intergenic region) (*14*). Those techniques amplify circulating DNA from peripheral-blood parasites in addition to DNA released from dead parasites (*15*) sequestered in capillaries or accumulating in extravascular spaces of the bone marrow and spleen, providing an indirect estimate of the total parasite burden harbored by the host.

### Parasite Sampling and Genotyping

To map the spread of parasite lineages across the region, we genotyped isolates collected during the panel study waves (Mâncio Lima sample set) along with 3 additional sample sets (Table 1; Appendix Figure 2). The Azul River/Nova Cintra set comprised samples obtained in April 2016 during cross-sectional surveys in riverine villages along Azul River, 6–8 hours by motorboat from the municipality of Mâncio Lima, and Nova Cintra on the Juruá River, municipality of Rodrigues Alves, in addition to samples collected in July 2018 and July 2019 along Azul River (*14*). The Vila Assis Brasil set included samples collected during cross-sectional surveys in August–September 2018, March 2019, and September–October 2019 in a periurban village 15 km away from Mâncio Lima by paved road (*16*). The health facility sample set consisted of isolates from symptomatic patients with microscopy-confirmed malaria who attended clinics in the municipalities of Mâncio Lima, Cruzeiro do Sul, and Rodrigues Alves during April 2018‒April 2020; most of those samples were collected in 2019 (*11*). We inferred the likely sites of infection by considering patients’ places of residence and history of recent travel.

**Table 1:** *Plasmodium* spp. samples used for molecular genotyping in study of microscopy sensitivity and decreased malaria prevalence in the urban Amazon Region, Brazil, 2018–2021

Species and year	Sample collection site	Place of infection, urban/periurban or rural	Symptoms, Y/N	Microscopy, positive/negative	Total
*P. vivax*					
2016	Azul River and Nova Cintra	0/2	0/2	0/2	2
2018	Mâncio Lima	42/0	5/37	7/35	42
	Vila Assis Brasil	0/42	9/33	11/31	42
	Health facilities	2/1	3/0	3/0	3
	All sites, 2018	44/43	17/70	21/66	87
2019	Mâncio Lima	28/0	2/26	1/27	28
	Vila Assis Brasil	0/61	6/55	4/57	61
	Azul River	0/3	1/2	1/2	3
	Health facilities	14/33	47/0	47/0	47
	All sites, 2019	62/77	56/83	53/86	139
2020	Mâncio Lima	13/0	0/13	1/12	13
2021	Mâncio Lima	23/0	4/19	1/22	23
Total, all sites, 2016–2021		122/142	77/187	76/188	264
*P. falciparum*					
2018	Azul River	0/2	0/2	2/0	2
	Mâncio Lima	39/0	15/24	17/22	39
	Vila Assis Brasil	0/10	2/8	4/6	10
	Health facilities	3/0	3/0	3/0	3
	All sites, 2018	42/12	20/34	26/28	54
2019	Azul River	0/3	0/3	0/3	3
	Mâncio Lima	7/0	2/5	1/6	7
	Vila Assis Brasil	0/19	3/16	1/18	19
	Health facilities	13/36	49/0	49/0	49
	All sites, 2019	20/58	54/24	51/27	78
2020	Mâncio Lima	9/0	1/8	0/9	9
	Health facilities	2/0	2/0	2/0	2
	All sites, 2020	9/2	3/8	2/9	11
2021	Mâncio Lima	19/0	4/15	0/19	19
Total, all sites, 2018-21		92/70	81/81	79/83	162

We typed 6 microsatellite loci for each species: MS2, MS5, MS6, MS7, MS9, and MS15 for *P. vivax* (*17*) and polyα, TAA81, TAA42, TA87, TA109, and TA60 for *P. falciparum* (*18*) (Appendix Table 1). We defined haplotypes as unique combinations of alleles at each locus, considering only the most abundant allele when >2 alleles were detected in the same sample (Appendix). We calculated the expected heterozygosity as an estimate of genetic diversity and the standardized index of association as a measure of multilocus linkage disequilibrium with LIAN 3.7 software as previously described (*19*) (Appendix). We used the goeBURST algorithm implemented in PHYLOViZ (https://www.phyloviz.net) to identify clusters of genetically related haplotypes (*20*) that might be informative about parasite migration patterns (Appendix Figure 3).

### Statistical Analysis

We entered data using the REDCap system (https://projectredcap.org/software) and analyzed using Stata 15.1 software (StataCorp LLC, https://www.stata.com). We defined statistical significance at the 5% level for 2-tailed tests. We calculated sensitivity, specificity, positive predictive value (PPV), negative predictive value (NPV), and accuracy, along with their 95% CIs, for microscopy and clinical signs and symptoms as predictors of infection detected by molecular methods (Appendix).

We used multivariable mixed-effects logistic regression models to identify correlates of malarial infection and disease while adjusting for potential confounders. We performed separate analyses for the following outcomes: TaqMan-confirmed *P. vivax,* regardless of any symptoms; TaqMan-confirmed *P. falciparum* infection, regardless of any symptoms; and clinical malaria (TaqMan-confirmed *P. vivax* or *P. falciparum* infection and reported fever, chills, or headache within the previous 7 days) (Appendix). We estimated odds ratios (ORs) with 95% CIs to quantify the influence of each predictor on the outcome. We also used logistic regression models (*21*) to describe the detectability of infection by microscopy as a continuous function of the number of species-specific amplicon copies per microliter measured by TaqMan.

## Results

### Prevalence of Submicroscopic and Asymptomatic Infection

We analyzed data from 2,774 residents in Mâncio Lima, ranging in age from 4 months to 103 years and distributed into 879 households, who underwent malaria parasite screening during >1 study wave. The number of participants varied from 1,093 in wave 1 to 2,043 in wave 7 (Appendix Table 2); 529 (19.1%) persons were screened at all time points, whereas 402 (14.5%) participated in a single study wave. Out of 11,730 specimens screened by PCR, 11,717 (99.9%) were also tested by microscopy.

PCR followed by TaqMan detected ≈10 times more infections than microscopy: out of 11,730 samples screened, 467 (4.0%) were positive for *Plasmodium vivax*, 104 (0.9%) for *P. falciparum,* and 54 (0.5%) for both species. Microscopy revealed 42 (0.4%) samples positive for *P. vivax*, 18 (0.2%) for *P. falciparum*, and 1 (0.01%) for both species, among 11,717 microscopically examined samples; we identified no other *Plasmodium* species (Table 2). None of the 54 mixed-species infections detected by TaqMan had been correctly identified by microscopy. Conversely, the only mixed-species infection diagnosed by microscopy yielded a positive TaqMan result for *P. vivax* only (Appendix Table 3).

**Table 2:** Factors associated with *Plasmodium* spp. infection and clinical malaria, as revealed by mixed-effects multiple logistic regression analysis, in the urban population of Mâncio Lima, Brazil, 2018–2021*

Factor	Outcome							
	*P. vivax* infection			*P. falciparum* infection			Clinical malaria	
	OR (95% CI)	p value		OR (95% CI)	p value		OR (95% CI)	p value
Individual-level variables								
Age								
<10	Referent			Referent	Ref		1	Ref
10–19	3.35 (1.99–5.67)	<0.0001		2.48 (0.97–6.33)	0.057		3.49 (1.01–12.11)	0.048
20–29	4.00 (2.22–7.19)	<0.0001		3.57 (1.38–9.25)	0.009		6.24 (1.82–21.46)	0.004
30–39	3.39 (1.99–5.80)	<0.0001		2.29 (0.90–5.83)	0.082		4.34 (1.31–14.41)	0.017
40–49	3.40 (1.83–6.33)	<0.0001		3.35 (1.21–9.26)	0.020		6.97 (2.16–22.53)	0.001
50–59	2.71 (1.38–5.31)	0.004		3.11 (1.10–8.81)	0.032		4.98 (1.27–19.53)	0.021
>60	3.94 (2.07–7.49)	<0.0001		3.74 (1.41–9.91)	0.008		8.56 (2.52–28.98)	0.001
Trend		0.003			0.011			<0.0001
Sex								
F	Referent			Referent			Referent	
M	1.04 (0.79–1.38)	0.767		1.72 (1.20–2.47)	0.003		0.72 (0.48–1.06)	0.095
Any past malaria								
No	Referent			Referent			Referent	
Yes	2.39 (1.70–3.34)	<0.0001		3.27 (1.93–5.52)	<0.0001		4.29 (2.58–7.12)	<0.0001
Bed net use past night								
No	Referent			Referent			Referent	
Yes, not treated	0.70 (0.48–1.02)	0.063		0.74 (0.40–1.34)	0.318		0.42 (0.23–0.76)	0.004
Yes, insecticide-treated	0.91 (0.67–1.23)	0.530		0.84 (0.52–1.37)	0.494		0.59 (0.35–1.00)	0.051
Household-level variables								
Wealth index quartile								
1, poorest	Referent			Referent			Referent	
2	0.60 (0.44–0.82)	0.001		0.53 (0.31–0.90)	0.019		0.52 (0.28–0.93)	0.028
3	0.46 (0.30–0.71)	<0.0001		0.63 (0.36–1.07)	0.092		0.52 (0.29–0.94)	0.029
4, wealthiest	0.39 (0.22–0.71)	0.002		0.51 (0.23–1.12)	0.094		0.39 (0.12–1.27)	0.118
Trend		<0.0001			0.064			0.018
Presence of eave gaps								
Yes	Referent			Referent			Referent	
No	0.71 (0.42–1.19)	0.194		0.47 (0.24–0.95)	0.034		0.11 (0.03–0.46)	0.002
Study wave number and dates								
1, Apr–May 2018	Referent			Referent			Referent	
2, Sep–Oct 2018	1.61 (1.05–2.49)	0.030		2.26 (1.17–4.34)	0.014		3.78 (1.91–4.50)	<0.0001
3, May–Jun 2019	1.50 (0.96–2.33)	0.073		2.29 (1.27–4.13)	0.006		2.15 (0.97–4.75)	0.058
4, Sep–Oct 2019	0.77 (0.48–1.25)	0.294		1.18 (0.56–2.49)	0.664		0.56 (0.20–1.60)	0.283
5, Oct–Nov 2020	0.54 (0.32–0.92)	0.025		0.68 (0.29–1.59)	0.378		0.48 (0.16–1.41)	0.183
6, Apr–May 2021	0.24 (0.13–0.42)	<0.0001		0.43 (0.17–1.05)	0.063		0.10 (0.01–0.83)	0.032
7, Oct–Nov 2021	0.26 (0.15–0.47)	<0.0001		0.34 (0.11–1.02)	0.053		0.56 (0.18–1.71)	0.308
No. observations†	11,676			11,615			11,569	

*Infection is defined as a positive genus-specific PCR result confirmed by species-specific TaqMan assay positive for *Plasmodium vivax* or *P. falciparum,* regardless of any symptoms. Clinical malaria is defined as a positive genus-specific PCR result confirmed by species-specific TaqMan assay positive for *P. vivax* or *P*. *falciparum*. in a study participant who reported symptoms (fever, chills, or headache) within the previous 7 days. Note that clinical malaria may be due to *P. vivax*, *P*. *falciparum*, or both species (mixed infections). OR, odds ratio. †The numbers of observations differ among outcomes because some exposure categories were dropped from the analysis in order to allow logistic models for *P. falciparum* infection and clinical malaria to converge.

Compared with molecular methods, microscopy demonstrated a diagnostic sensitivity of 8.5% (95% CI 6.5%–11.1%), specificity of 99.9% (95% CI 99.9%–100.0%), PPV of 86.9% (95% CI 75.9%–93.3%), NPV of 95.1% (95% CI 95.0%–95.2%), and accuracy of 95.1% (95% CI 94.7%–95.5%) (Appendix Tables 3, 4). The proportion of molecularly diagnosed malarial infections that were asymptomatic varied from 73.8% in wave 2 to 96.8% in wave 6, with a higher proportion of asymptomatic infections during the latest study waves (Table 2). When compared with PCR followed by Taqman, the overall diagnostic sensitivity of reported fever, chills, or headache was only 16.0% (95% CI 13.2%–19.1%); specificity was 90.8% (95% CI 90.3%–91.4%), PPV 8.9% (95% CI 7.53%–10.6%), NPV 95.1% (95% CI 94.9%–95.2%), and accuracy 86.8% (95% CI 86.2%–87.5%). Overall, we observed a higher prevalence of infection with *P. vivax* and *P. falciparum* and of clinical malaria among adolescent and adults from the poorest households in the town (Appendix Tables 2,5), as indicated by adjusted multiple logistic regression analysis incorporating >11,000 observations.

### Parasite Detectability over Time

The prevalence of malarial infection diagnosed by microscopy and molecular methods, either symptomatic or asymptomatic, significantly declined during the study period (Figure 2; Appendix Table 2). The later study waves exhibited an increased proportion of submicroscopic infections, which might be attributed to lower average parasite densities during periods of decreased transmission (*21*). However, TaqMan-quantified *P. vivax* densities remained consistent during 2018–2021 at an overall geometric mean of 19.0 amplicon copies/μL (Appendix Table 2). In contrast, *P. falciparum* densities (geometric mean 10.5 amplicon copies/μL) fluctuated more prominently, from 3.2 amplicon copies/μL in wave 6 to 86.3 amplicon copies/μL in wave 5, although the limited sample size prevented robust identification of temporal trends (Appendix Table 2).

**Figure 3:**
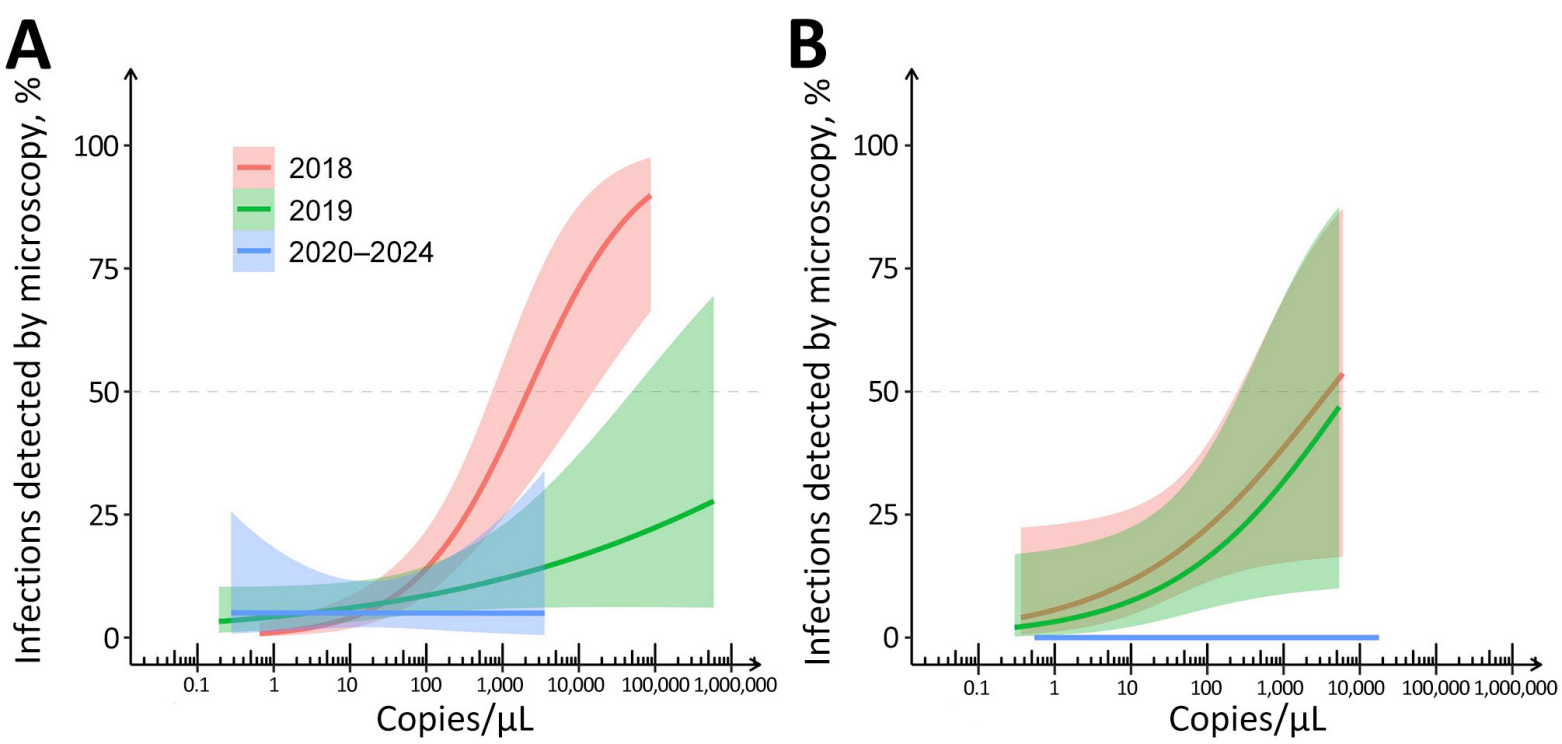
Proportion of TaqMan*-*detected single-species *Plasmodia vivax* (A) and *P. falciparum* (B) infections that were detected by microscopy according to parasite density (amplicon copies per microliter measured by species-specific TaqMan assays) in study of microscopy sensitivity and decreased malaria prevalence in the urban Amazon Region, Brazil, January 2018–December 2021. Lines represents the fitted logistic model trends; the shaded area indicates 95% CI for waves 1 and 2 (2018; red), waves 3 and 4 (2019; green) and waves 5–7 (2020–2021; blue). For this analysis, data from waves 1 and 2 (2018), 3 and 4 (2019), and 5–7 (2020–2021) were combined to achieve balanced sample sizes for fitting logistic models. CI could not be properly estimated for *P. falciparum* infections in waves 5–7 because of the small sample size (Appendix Table 2). The dashed horizontal line indicates 50% microscopic detectability at a given parasite density threshold, which for *P. vivax* infections in 2018 corresponds to 2,088 (95% CI 734‒14,572) amplicon copies/μL.

The likelihood of detecting parasite by microscopy as a function of TaqMan-measured parasite density diminished with time. In 2018, we anticipated to identify 50% of *P. vivax* infections by microscopy at a density of 2,088 (95% CI 734–14,572) amplicon copies/μL or ≈42 parasites/μL (Figure 3, panel A). Nevertheless, most *P. vivax* infections above this threshold were undetected by microscopy in subsequent years, consistent with a decline in microscopy sensitivity. At a given TaqMan-measured average parasite density, the diagnostic sensitivity of peripheral-blood microscopy decreased during periods of lowest malaria prevalence. Despite the considerably smaller sample size, we noted a similar temporal trend in the microscopic diagnosis of *P. falciparum* infections (Figure 3, panel B). Those findings suggest that a higher proportion of infections are overlooked by peripheral-blood microscopy as malaria prevalence declines.

### Parasite Lineages over Space and Time

**Figure 4:**
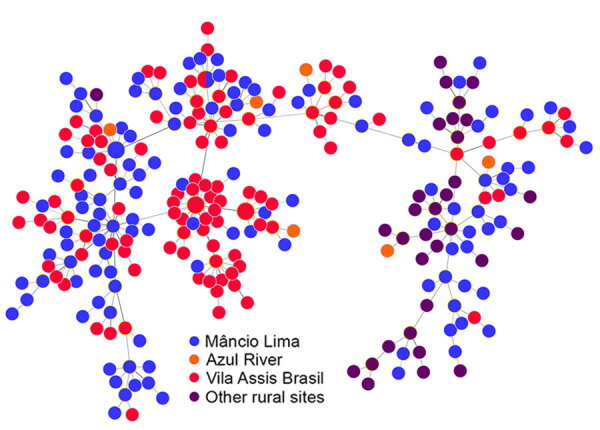
Minimal spanning trees representing the connectivity of *Plasmodium vivax* haplotypes from the Juruá Valley region of Brazil (264 isolates collected 2016‒2021). Circles represent haplotypes with size linearly proportional to the number of isolates sharing them. Lines connect pairs of haplotypes with <5 allele mismatches and the overall network represents the most likely haplotype genealogy, ensuring that the summed distance of all links of the tree is the minimum possible. Haplotype colors indicate the likely site of infection (Appendix Figure 2). The circle with blue and red slices indicates a haplotype that was shared by 2 parasites from different (urban and rural) origins.

**Figure 5:**
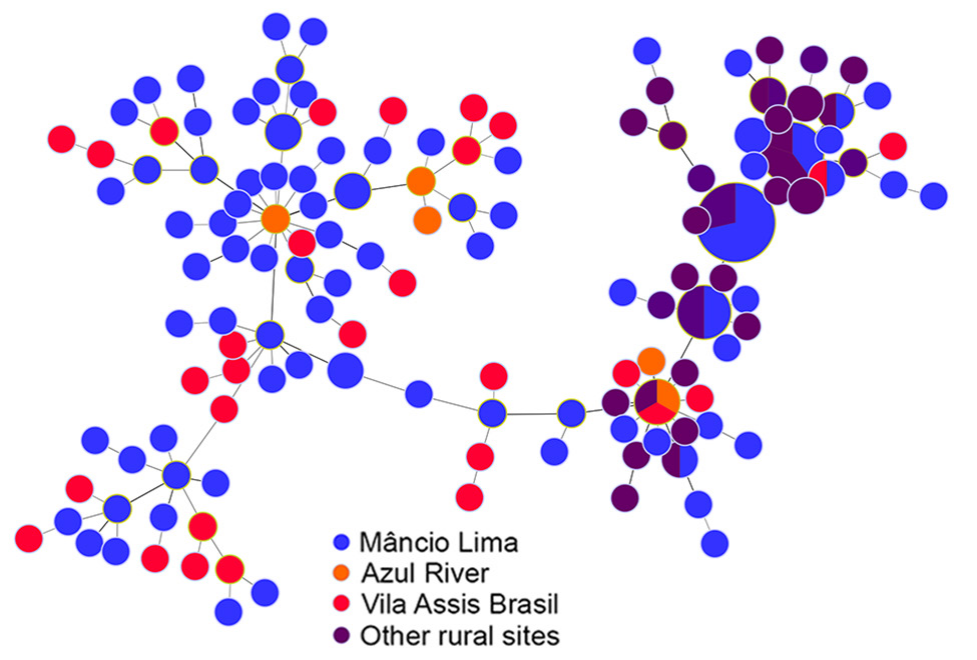
Minimal spanning trees representing the connectivity of *Plasmodium falciparum* haplotypes from the Juruá Valley region of Brazil (162 isolates collected 2018 ‒2021). Similar to Figure 4, circles represent haplotypes with size linearly proportional to the number of isolates sharing them and lines connect pairs of haplotypes with <5 allele mismatches. Haplotype colors indicate the likely site of infection (Appendix Figure 2). In 7 circles (haplotypes), slices of different colors indicate that the corresponding haplotypes were shared by parasites from different geographic origins.

During 2018–2021, we genotyped 122 *P. vivax* and 92 *P. falciparum* isolates collected in the town of Mâncio Lima, along with 142 *P. vivax* and 70 *P. falciparum* isolates from surrounding rural areas (Table 1; Appendix Figure 2). The parasites exhibited a high genetic diversity (average heterozygosity >0.8 for both species) that varied little over time in Mâncio Lima (Appendix Table 2). Both *P. vivax* and *P. falciparum* populations displayed substantial linkage disequilibrium (Appendix), consistent with the circulation of near-clonal parasite lineages across the region (*22*). Of interest, minimal spanning trees showed no clear clustering based on infection sites in the Juruá Valley for *P. vivax* (Figure 4) and *P. falciparum* (Figure 5). Instead, parasite haplotypes from the town of Mâncio Lima and the rural surroundings were evenly distributed in the trees. We observed 1 instance for *P. vivax* and 6 instances for *P. falciparum* of haplotype sharing among parasites from the town and rural areas, indicating substantial connectivity between urban and rural populations.

We observed some evidence of temporal clustering of parasite lineages (Appendix). Parasites from both species circulating in the town of Mâncio Lima during 2019 formed clusters (Appendix Figure 4) that were enriched in lineages from symptomatic and patent infections (Appendix Figure 5). Similarly, clusters of patent and symptomatic *P. vivax* and *P. falciparum* infections were also observed in the analysis of the complete Juruá Valley dataset, combining data from urban and rural sites (Appendix Figure 6).

## Discussion

In this extensive population-based study spanning 4 years, we found increasing proportions of asymptomatic and submicroscopic infections as the overall number of urban malaria cases decreased in the main transmission hotspot of Brazil. Of notable practical importance, the utility of microscopic diagnosis for detecting parasitemia diminished as malaria transmission decreased, which carries medical and public health implications. This challenge is not unique to our study site; microscopy has been demonstrated to overlook a higher proportion of *P. falciparum* infections in the areas of lowest malaria prevalence across sub-Saharan Africa (*23*). Molecular diagnosis may be necessary for identifying low-density malarial infections in low transmission settings, where microscopy-based parasite prevalence is <10% (*24*). However, new field-deployable tests for parasite DNA detection should undergo validation before widespread implementation in peripheral laboratories in the Amazon Basin (*5*). Furthermore, positive PCR results should be interpreted as indicative of either current or recent infection (*15*).

Few studies have identified changes in microscopy sensitivity with decreasing malaria transmission by repeatedly sampling the same population (*16*,*25*). Here, we provide evidence of rising microscopy detection thresholds, showing that a higher proportion of *P. vivax* and *P. falciparum* infections were missed at comparable parasite densities as malaria incidence diminished over time. Of note, molecular methods can detect circulating DNA released by dead trophozoites and schizonts that may have been concealed in capillaries and venules or extravascular territories (*15*). We speculate that, at lower transmission, fewer parasites accumulating in deep-organ blood vessels and extravascular spaces tend to circulate in the peripheral blood, where they can be identified by microscopy, perhaps indicative of parasites’ adaptation to seasonal variation in vector abundance (*26*) or enhanced control measures (*27*). Temporal changes in *P. falciparum* sequestration patterns, particularly in regions with seasonal malaria transmission (*26*), can theoretically influence parasite detectability in the peripheral blood. However, additional longitudinal studies are required to validate this hypothesis.

Our findings demonstrate that parasite lineages spread across rural‒urban boundaries, suggesting that rural areas with limited diagnosis and surveillance may serve as important sources for malaria reintroduction. Rural‒urban mobility introduces malaria parasites into receptive, more densely populated urbanized spaces (*28*). The most mobile residents of Mâncio Lima are men 16‒60 years of age who lack formal employment in the town and are often engaged in subsistence or commercial farming in periurban settlements (*29*). We found that closely related lineages of *P. vivax* and *P. falciparum* are shared among urban and rural residents, indicating the free circulation of malaria parasites across the rural‒urban boundaries in the Juruá Valley. Efficient parasite spreaders, such as forestgoers in Southeast Asia villages (*30*), can be selectively targeted for more intensive and effective malaria control interventions that cannot be readily delivered to the entire community, in this and similar endemic settings across the Amazon.

Brazil has an ambitious plan to achieve zero malaria by reducing cases from 150,000 in 2022 to <68,000 by 2025, eliminating *P. falciparum* malaria and associated deaths by 2030, eliminating cases and deaths by any species by 2035, and preventing malaria reintroduction from 2035 onward (*31*). However, elimination plans can be undermined by the establishment of malaria transmission in highly connected urbanized spaces that serve as both sinks and sources of parasite lineages. The large urban and periurban malaria outbreaks recorded in the Juruá Valley since the mid-2000s, when the region became the main transmission hotspot of Brazil, have been linked to the opening of fishponds for commercial aquaculture (*6*,*7*,*32*). By 2015, the Juruá Valley contributed 18% of the country’s malaria burden (*33*). Although most infections diagnosed during our study were asymptomatic, parasite carriers are exposed to recurrent episodes of parasitemia, which lead to chronic anemia and other possible clinical and immunologic consequences (*34*). Of importance, the hidden parasite reservoir missed by routine surveillance substantially contributes to human-to-mosquito parasite transmission in similar low-endemicity settings (*4*,*21*,*35*,*36*).

The country’s strategy for malaria surveillance and control comprises several actions: to provide prompt microscopy-based diagnosis and treatment after detection of isolated cases or clusters of cases; to detect additional cases, once an index case has been diagnosed; and to implement vector control measures in the vicinity of passively detected cases (*37*). Active surveillance around cases has gradually been implemented in Brazil since reactive case detection was demonstrated to be feasible and effective for malaria control in the Amazon (*38*), although molecular diagnosis is rarely used with this purpose.

The role of long-lasting insecticide-treated bed nets (LLINs) and indoor residual spraying in malaria control in Mâncio Lima is uncertain. Only 46% of the study participants reported using LLINs the previous night; <15% had their houses sprayed within the previous 12 months (Appendix Table 5). Larval source management is currently limited to periodically removing vegetation from the margins of fish farming tanks and emptying those no longer used for aquaculture. Alternatively, the periodic application of environmentally safe biolarvicides can drastically reduce anopheline larval density in fish tanks (*39*), but its feasibility for large-scale use remains to be determined.

The primary strength of this study lies in its household panel design (*12*), which enabled us to examine temporal trends of malarial infection and disease at the community level and to identify changes in the sensitivity of diagnostic microscopy over 4 years. The first limitation of this study is that only 61 microscopy-positive infections and 100 clinical malaria episodes were diagnosed during 7 study waves, despite screening a large population, as expected under low transmission. Second, we defined clinical malaria on the basis of perceived signs and symptoms, rather than fever measured at the time of interviews. We argue that perceived symptoms, although not highly specific and prone to recall bias, are the key drivers of healthcare seeking and define which infections will be identified by passive surveillance and eventually treated. Third, microsatellite genotyping provides a relatively limited view of genetic variation and connectivity among local parasites, compared with targeted amplicon sequencing (*40*) or whole-genome analysis (*41*). However, our results are broadly consistent with genome-wide analyses that showed recent ancestry sharing between urban and rural near-clonal lineages of *P. vivax* across Juruá Valley (*22*).

In conclusion, our research illustrates how parasite lineages can spread across rural‒urban boundaries and cause predominantly asymptomatic infections that are increasingly missed by microscopy as the overall malaria incidence drops. The presence of an undetected urban malaria reservoir may require more effort toward regional control and elimination strategies.

Appendix Additional information about microscopy sensitivity and decreased malaria prevalence in the urban Amazon Region, Brazil, 2018–2021.
