# Supplementary material for: Lower Microscopy Sensitivity with Decreasing Malaria Prevalence in the Urban Amazon Region, Brazil, 2018–2021
Source: Emerg Infect Dis. 2024 Sep;30(9):1884–94. doi: 10.3201/eid3009.240378 (PMC11346994; doi:10.3201/eid3009.240378)
Supplement: Appendix — Additional information about microscopy sensitivity and decreased malaria prevalence in the urban Amazon Region, Brazil, 2018–2021. [file 24-0378-Techapp-s1.pdf]

Article DOI: https://doi.org/10.3201/eid3009.240378

EID cannot ensure accessibility for supplementary materials supplied by authors. Readers who have difficulty accessing supplementary content should contact the authors for assistance.

Lower Microscopy Sensitivity with Decreasing Malaria Prevalence in the Urban Amazon Region, Brazil, 2018–2021

Appendix

Appendix Methods

Study Area

The municipality of Mâncio Lima, located in the upper Juruá Valley region of Acre State, westernmost Brazil, has an equatorial climate, with an average annual temperature of 26°C and average annual precipitation of 2,167 mm, with most rains between November and April. The municipality of Mâncio Lima has a single urban area, distributed from the Southeast to the Northwest along the BR-364 highway, next to the Japiim river (7°36′28.6” S, 72°54′23.0” W; Appendix Figure 1). At the study onset, in 2018, 48.7% of malaria cases in the Municipality of Mâncio Lima were reportedly acquired in the urban area. There are eight government-run laboratories operating in health care facilities in the urban area of Mâncio Lima that provide free microscopy-based malaria diagnosis. *Plasmodium vivax* accounts for 84.2% of locally acquired laboratory-confirmed infections; 14.4% are due to *P. falciparum* and 1.4% to both species (*1*).

During the baseline population census conducted by our team in the town of Mâncio Lima between November 2015 and April 2016, we enumerated 9,124 permanent residents, with ages ranging between <1 month and 105 years, distributed into 2,329 households (*2*). The northern part of the municipality is covered by rainforest, with some forest fragments remaining in the south (Supplementary Figure 1). The nearby forest is seasonally flooded, and the town is crossed by small streams. Agriculture and livestock production are the main economic activities in Mâncio Lima; formal jobs are scarce in the urban area and mainly restricted to activities related to public administration. As a consequence, a large proportion of the urban population is

involved in typically rural activities, such as commercial or subsistence farming, which greatly encourages urban-rural mobility.

Defining “Urban Area” in the Amazon

Following decades of massive rural-to-urban migration, 75% of the population of the Brazilian Amazon currently lives in settlements defined as cities or towns by the Brazilian Institute of Geography and Statistics (Portuguese acronym, IBGE) (https://censo2022.ibge.gov.br/panorama/). Importantly, 29% of urban growth in the region was in informal occupations such as shanty towns, which lack a properly urban infrastructure (https://brasil.mapbiomas.org/en/2022/11/04/favelas-no-brasil-crescem-em-ritmo-acelerado-e-ocupam-106-mil-hectares/).

However, among epidemiologists and malariologists there is no consensus regarding the definition of “urban” (*3*). In Brazil, the 1988 Federal Constitution and Federal Law 10.257/01 mandate that municipalities are responsible for defining the boundaries of their urban area. The Brazilian Institute of Geography and Statistics (Portuguese acronym, IBGE), in turn, adopts the classification defined by each Brazilian municipality. No minimum total population or population density is required to classify a settlement as urban in Brazil and some towns have less than 1,000 inhabitants (https://www.cnnbrasil.com.br/nacional/censo-2022-veja-as-10-maiores-e-menores-cidades-do-pais/).

Urbanized spaces are particularly difficult to define in the Amazon, where cities and towns are connected to surrounding frontier settlements, traditional riverine villages, and even indigenous communities, through a process termed extended urbanization (*4,5*). Even apparently less urbanized areas are often affected by the production structure, consumption, and even lifestyle of urban areas (*6*). In this study, we delineated the urban area of the Municipality of Mâncio Lima essentially as IBGE does, but we considered the qualitative “urbanicity” criteria of Dal'Asta and colleagues (*7*) to extend the urban area to two additional neighborhoods (namely, Iracema and Pé da Terra) situated along the main road that crosses the town (*2*).

Study Population

We analyze data and biologic samples from the Mâncio Lima Cohort Study of Urban Malaria (ClinicalTrials.gov registration number, NCT03689036), which comprises ≈20% of the residents in the town of Mâncio Lima (*2*). We used simple probability sampling to draw ≈20%

households in the urban area of Mâncio Lima from our census listing. Next, we invited all residents in 534 randomly selected households to participate in the baseline survey, in April-May 2018 (wave1); 1,391 residents from 354 households (15.2% of those enumerated in the census) were located and interviewed face to face in their residences. To achieve the desired sample size of 20%, 147 "substitute" households were randomly selected and approached during the second visit, along with the original household sample, in September-October 2018 (wave 2). The subsequent study waves were in May-June 2019 (wave 3), September-October 2019 (wave 4), October-November 2020 (wave 5), April-May 2021 (wave6), and October-November 2021 (wave 7). The original study design comprised semiannual waves, but that planned for April-May 2020 did not occur because of the COVID-19 pandemic.

Here, we used a household panel design for data analysis. Study participants leaving the sampled households were retained in the panel survey, as long as they could be located by the field team and their new residences, which were labeled as new households, were situated in the urban area of Mâncio Lima. The new residents ("entrants") in originally sampled households were invited to participate. As a consequence, the total number of households in the sample has increased since the second wave. Some study participants could not be located during a visit but were later "rescued" during the next waves. Participants who died, moved away from the study site, and those who withdrew their consent to participate were considered lost for follow-up (*2*).

Sociodemographic and morbidity information was collected and updated during each study wave. Data on household assets were combined to derive a household-based wealth index – a proxy of socioeconomic status (*8*). Capillary blood samples were collected by finger prick from consenting participants >3 months old for malaria diagnosis and parasite genetic studies, regardless of any symptoms (*2*). To characterize clinical malaria, participants were first asked whether they had any signs or symptoms that might have been caused by a malarial infection. Those reporting signs or symptoms at or up to 7 days before the interview were specifically asked whether they had fever, chills, or headache – the main clinical manifestations of uncomplicated malaria in semi-immune Amazonians (*9*).

Rapid Diagnostic Tests for Malaria Antigen Detection

During waves 3 (May-June 2019) and 4 September-October 2019), we used the QuickProfile Malaria Pf/Pv Antigen rapid diagnostic test (RDT) produced by Lumiquick

Diagnostics (Santa Clara, CA), in addition to conventional microscopy, for on-site malaria diagnosis. This RDT targets the *P. falciparum*-specific histidine rich protein 2 (HRP2) and the *P. vivax*-specific lactate dehydrogenase (LDH) and meets the World Health Organization prequalification requirements (*10*) The detection threshold was estimated by the manufacturer as 100 parasites/µL of blood for *P. falciparum* and 150 parasites/µL for *P. vivax* (https://lumiquick.co/infectious-diseases-1).

Molecular Diagnosis of Malaria

Capillary blood aliquots kept at −20°C were shipped to our research laboratory in São Paulo for molecular screening for malaria. To this end, parasite DNA was extracted from 50 µL of whole blood using DNA Investigator kits (Qiagen, Hilden, Germany) and an automated QIASymphony platform (Qiagen). The final DNA elution volume was 100 µL. Samples were considered positive for malaria parasites by nucleic acid amplification if they had a positive SYBR Green-based genus-specific PCR result and had DNA from either *P. vivax*, *P. falciparum*, or both species, subsequently amplified by the species-specific, quantitative TaqMan assays.

The genus-specific screening PCR used the oligonucleotide primers PCBF (5′-ATG CTT TAT TAT GGA TTG GAT GTC-3′ and PCBR (5′-CAG ACC GTA AGG TTA TAA TTA TGT-3′) to amplify a conserved sequence of the *cytochrome b* (*cytb*) gene of human-infecting malaria parasites (*11*). The 20-µL reaction contained 5 µl of DNA solution, 7.5 µL of 2× Maxima SYBR Green quantitative PCR master mixture (Fermentas, Burlington, Canada), and 0.3 µM of each primer. The amplification protocol comprised a 2-min step at 50°C, followed by 10-min denaturation step at 95°C and 40 cycles (95°C for 15 s and 60°C for 1 min) on a QuantStudio 6 real-time PCR system (Thermo Fisher Scientific, Waltham, MA, USA). The PCR detection threshold was estimated at 0.2 amplicon copies per µL, corresponding to ≈4 parasites per mL, assuming an average of 50 mitochondrial genome copies per blood-stage parasite, by serially diluting a plasmid preparation containing the target sequence at a known DNA concentration. No-template negative controls, containing all reagents for amplification except for the DNA template, were run for every PCR microplate.

PCR-positive samples were further tested with newly designed TaqMan assays that target species-specific mitochondrial genome sequences (*12*). The *P. vivax* protocol used the primers 5′-TTT GGT GGT ACT ACA GGA GTA ATA TTA GGT-3′ and 5′-GAA ATG AGC GAT

TAC ATA GTA AGT ATC ATG-3′ and the probe 5′-VIC-TGC AGC TAT TGA TAT TGC AT-MGB (minor groove binder)-NFQ (Applied Biosystems proprietary quencher)-3′ to amplify the target fragment size of 84 bp [bp] at the *cox1* gene (positions 3806–3885 of the complete mitochondrial genome of the South Korea A2 isolate, GenBank accession number NC_028626.1). The *P. falciparum* protocol used the primers 5′-CAT TAT GAT TAC AGC TCC CAA GCA-3′ and 5′-GGT CTG ATT TGT TCC GCT CAA TA-3′ and the probe 5′ FAM -TAC AAG ATT GTG ATA AGA TGA C- MGB-NFQ–3’ to amplify a 90-bp target fragment comprising the 3′ end of the *cox1* gene and an intergenic region (positions 4590–4679 of the complete mitochondrial genome of the K1 isolate, GenBank accession number NC_037526.1). Each 20-µL reaction for both assays, carried out in 96-well microplates, contained 5 µL of DNA solution and 10 µL of 2 TaqMan Universal Mater Mix II, no-UNG (Thermo Fisher Scientific). In the *P. vivax* protocol, we used 0.2 µM of each primer and 0.1 µM of the probe per reaction; in the *P. falciparum* protocol, we used 0.1 µM of each primer and 0.08 µM of the probe. The amplification cycles were identical for both species and comprised a 2-min step at 50°C, followed by a 10-min denaturation at 95°C and 40 cycles at 95°C for 15 s and at 60°C for 1 min, carried out on a QuantStudio 6 real-time PCR system (Thermo Fisher Scientific). Standard curves were prepared with nine serial tenfold dilutions of the respective target sequences and tested in each microplate to allow for species-specific quantitation of parasite loads (number of amplicon copies/µL of blood). The limits of detection were estimated at 1 amplicon copy per µL (≈20 parasites per mL) for *P. vivax* and 10^{-3} amplicon copies per µL (≈0.2 parasites per mL) for *P. falciparum* (*12*), assuming an average of 50 mitochondrial genome copies per blood-stage parasite.

Malaria Treatment

Plasmodium vivax infections diagnosed by on-site microscopy were treated with chloroquine (25 mg/kg over 3 days) and primaquine (3.5 mg/kg over 7 days); *P. falciparum* infections were treated with a 3-day course of artemether (2 to 4 mg/kg/day) plus lumefantrine (12 to 24 mg/kg/day) and a single dose of 0.75 mg/kg primaquine for gametocyte clearance (*13*). Quality-assured, free antimalarials were provided by the local malaria program and the first-dose intake was supervised by the field team. Both treatment regimens remain highly efficacious in the area (*14*,*15*).

Parasite Genotyping

To map the circulation of parasite lineages across the Juruá Valley area, we genotyped parasites from TaqMan-positive samples collected between 2016 and 2021 in the town of Mâncio Lima and surrounding rural sites in the municipalities of Mâncio Lima, Rodrigues Alves, and Cruzeiro do Sul (main text, Table 1). The sites of infection are shown in Appendix Figure 2. We used 6 single-copy microsatellite loci (MS2, MS5, MS6, MS7, MS9, and MS15) that map to 5 different chromosomes of *P. vivax* (*16*) and 6 single-copy markers (polyα, TAA81, TAA42, TA87, TA109, and TA60) that map to 4 different chromosomes of *P. falciparum* (*17*). MS2 has tetranucleotide repeats, while all other markers comprise trinucleotide repeats (*16,17*). Microsatellite alleles were PCR amplified as previously described (*18*). PCR products were separated by capillary electrophoresis on an automated ABI 3730 DNA sequencer (Applied Biosystems) and their lengths (in base pairs (bp) and relative abundance (peak heights in electropherograms) were determined using GeneMarker 2.7 (Soft Genetics, State College, PA) software. The minimal detectable peak height was set to 900 fluorescence units. Alleles were defined according to amplicon sizes. We scored two alleles at a locus when the minor peak was >33% the height of the predominant peak, but considered only the major peak in subsequent analyses. Haplotypes were defined as unique combinations of alleles at each locus analyzed, considering only the most abundant allele for haplotype assignment when two or more alleles were detected in the same sample (i.e., in cases of multiple-clone infections) (*18*).

We used LIAN 3.7 software (*19*) to calculate the expected heterozygosity (H_E) as a measure of genetic diversity per marker, defined as $H_E = [n/(n-1)][1 - \Sigma p_i^2]$, where n is the number of isolates analyzed and p_i is the frequency of the i-th allele in the population. H_E gives the average probability that a pair of alleles randomly obtained from the population is different and ranges between 0 and 1. Average H_E was calculated by averaging H_E estimates obtained for each marker.

The standardized index of association (I_A^S) was calculated with LIAN 3.7 software (http://guanine.evolbio.mpg.de/cgi-bin/lian/lian.cgi.pl) as a measure of overall multilocus linkage disequilibrium (LD) in the parasite population. I_A^S compares the observed variance (V_D) of the number of alleles at which each pair of haplotypes differ in the population with the variance expected under random association of alleles (V_E) as follows: $I_A^S = (V_D)/ V_E) - 1)(1/l - 1)$, where l is the number of loci analyzed (n = 6) (*20*). V_E was derived from 10,000 random permutations

with LIAN 3.7 software (*14*). LD is expected to increase V_D relative to V_E (*20*); significant LD was detected whenever V_D was greater than 95% of V_E values. We used “clone-censored” databases containing only unique haplotypes (i.e., counting each shared haplotype only once) to rule out an apparent LD due to the occasional expansion of haplotypes in an otherwise panmictic population.

We calculated the pairwise number of different alleles or mismatches, out of 6 loci analyzed for each species, as an estimate of genetic distance between isolates. We used the goeBURST algorithm (*21*) implemented in PHYLOViZ 2.0 software (*22*) to represent haplotype genealogies as minimum spanning trees (MSTs). The goeBURST algorithm generates hypothetical haplotype genealogies by assuming that a founder haplotype propagates in the population and gradually diversify, as a result of mutation or recombination, to originate clusters of genetically related haplotypes that can still be traced to their ancestral haplotype (*21*). The hypothetical phylogenetic relationships between haplotypes are illustrated as an MST that connects all haplotypes with ≤5 allele mismatches in the sample in such a way that the summed distance of all links of the tree is the minimum (*21*).

We applied a simple molecular epidemiology framework to explore the relative contribution of parasites of urban versus rural origin to residual malaria transmission in Mâncio Lima (*23*). Briefly, we considered two hypothetical source-sink scenarios: (a) cases diagnosed in urban residents are exclusively imported from rural villages and (b) cases diagnosed in urban residents may be either imported or locally acquired (Appendix Figure 3).

In the first scenario, the town is purely a sink, represented as the large circle with interrupted contour in the center, and the rural villages (three smaller circles in the periphery) are the sources of parasites. Arrows indicate parasite mobility between locations and self-loops indicate parasite transmission within each site. Parasite lineages from each village (represented by different shapes with the same color) are introduced into the town but are not further transmitted locally, as indicated by the absence of a self-loop. Clusters of closely related parasites are occasionally found in the town, as different people may have been infected in the same source village (single-source outbreaks), but most parasites circulating among urban residents are not expected to be highly connected to each other in the absence of urban transmission. Moreover, parasites from different source villages (shapes with different colors)

may not be connected to each other if there is little human mobility among these rural sites. Therefore, under this scenario parasite lineages are expected to cluster according to their source location, with few, if any urban transmission networks.

In the second scenario, parasite lineages freely circulate across the region and each site is both a source and a sink of infection. Haplotypes from rural villages (different shapes within the three small circles in the periphery of the figure) are introduced into the town (large circle with continuous contour in the center) and further transmitted locally among urban residents, as indicated by the presence of a self-loop. As a result, haplotype networks comprise clusters of parasites from both urban and rural residents and from all putative source populations. Human mobility also introduces into rural villages some parasite lineages that originate from another rural village but circulate in the town. Therefore, imported lineages (shapes of different colors) are occasionally seen in the rural villages.

Statistical Analysis

Data were entered using REDCap (*24*) and analyzed with Stata version 15.1 software (Stata, College Station, TX, USA). Summary statistics are presented along with 95% confidence intervals (CIs); statistical significance was defined at the 5% level (two-tailed tests). We also calculated, for both microscopy and RDT, their sensitivity (probability that microscopy or RDT will be positive when the reference method yields a positive result), specificity (probability that microscopy or RDT will be negative when the reference method yields a negative result), positive and negative predictive values (PPV and NPV; probability that the reference test will be positive/negative when microscopy or RDT is positive/negative) and accuracy (probability that a sample will be classified in the same way by microscopy or RDT as the reference method) compared with the reference method (either PCR followed by TaqMan or microscopy), along with their 95% CIs. The same parameters were calculated for symptoms as a predictor of infection detected by molecular methods.

We entered observations from all seven cross-sectional surveys into multivariable logistic regression models to identify independent correlates of malarial infection and disease, while adjusting for potential confounders. Separate analyses were run for three outcomes: (1) TaqMan-confirmed *P. vivax,* regardless of any symptoms; (2) TaqMan-confirmed *P. falciparum* infection, regardless of any symptoms; and (3) clinical malaria (TaqMan-confirmed *P. vivax* or *P.*

falciparum infection and reported fever, chills, or headache within the past 7 days). No attempt was made to distinguish between imported infections and those acquired in the town of Mâncio Lima. Individual-level ("indvar") and household-level ("housevar") covariates were introduced in the models following a stepwise forward approach and maintained in the final models if they were significantly associated with at least of the outcomes. Individual-level variables were: sex, age (categorized as 0–9, 10–19, 20–29, 30–39, 40–49, 50–59, and ≥60 years), bed net use in the previous night (no; yes, untreated; yes, insecticide-treated [LLIN]), and one or more previous laboratory-confirmed malaria attacks reported by the participant (yes/no). Household-level variables were: wealth index (stratified into quartiles), house characteristics (wall material, presence of wall and eave gaps, and location of toilet/bathing facilities), and whether the house had been treated with indoor residual spray (IRS) in the past 12 months (yes/no).

We used the "xtset" Stata command to declare data to be fully nested panel data, with the panel variable corresponding to participants′ unique identifier and the time variable *wave* corresponding to the wavenumber (1 through 7). We note that the panel is "unbalanced" because not all individuals participated in all surveys. Due to the nested structure of the data, we used the "melogit" Stata command to build mixed-effects logistic regression models that included the grouping variables as random factors. We had repeated observations for most participants, who were clustered within households. We used the following Stata syntax: *melogit outcome indvar1 indvar2 housevar1 housevar2 wave|| household: || id: vce(robust) or*. Up to seven observations per individual were entered in multiple logistic regression analysis. Odds ratio (OR) estimates are provided along with 95% CIs to quantify the influence of each predictor on the outcome.

Appendix Results

RDT Compared with Molecular Methods and Microscopy

The comparison of the diagnostic sensitivity of microscopy and PCR followed by TaqMan is presented in the main text and Appendix Table 3. Next, we compared results obtained with the QuickProfile Malaria RDT during study waves 3 and 4 with those by molecular methods and microscopy (Appendix Table 4). Compared with molecular methods, the RDT had a diagnostic sensitivity of 3.8% (95% CI, 1.8%–7.1%), specificity of 99.8% (95% CI, 99.5%–99.9%), PPV of 56.2% (95% CI, 32.8%–77.4%), NPV of 93.2% (93.0%–93.4%), and accuracy

of 93.0% (92.1%–93.9%). Appendix Table 5 shows the comparison between results obtained with the RDT and conventional microscopy. The RDT had a diagnostic sensitivity of 38.1% (95% CI, 18.1%–61.6%), specificity of 99.8% (95% CI, 99.5%–99.9%), PPV of 50.0% (95% CI, 29.3%–70.0%), NPV of 99.6% (99.4%–99.7%), and accuracy of 99.4% (99.0%–99.6%), taking microscopy as the reference test.

We note that 46.5% of the *P. falciparum* isolates from the Juruá Valley in Brazil carry the *hrp2* gene deletion (87 of 187 samples examined) (*25*) – the highest prevalence of *hrp2* gene deletion observed in *P. falciparum* populations worldwide. Because the QuickProfile Pf/Pv Antigen RDT targets HRP2, the high proportion of false-negative results for *P. falciparum* observed in our study is unsurprising. These results highlight the low sensitivity of HRP2-based RDTs for falciparum malaria diagnosis in the region.

Correlates of Risk of Infection and Disease

Covariates significantly associated with the risk of infection with *P. vivax* and *P. falciparum* and clinical malaria in the study population are shown in Appendix Table 6 (unadjusted analysis) and Table 3, main text (fully adjusted multiple logistic regression models that combine >11,000 observations). The risk of infection and clinical malaria was elevated among adolescents and adults, compared with children <10 years, and among participants who reported one or more previous laboratory-confirmed malaria episodes, compared with malaria-naïve people. Male study participants were at increased risk of *P. falciparum* infection, compared with females. Having slept the past night under a bed net was a correlate of protection from disease, but not necessarily from infection. Interestingly, insecticide-untreated bed nets, which are widely available in local stores, appeared to confer greater protection than long-lasting insecticide-treated bed nets (LLINs) delivered by the malaria control program. This is most likely due to residual confounding, as LLIN distribution is selectively targeted to households at greater risk of infection, rendering its protective effect harder to detect. Residents in the poorest households were the most affected by *P. vivax* infection and disease, and those people living in houses with open eave gaps were at increased risk of clinical malaria (Table 3, main text).

Genetic Diversity in *Plasmodium vivax* and *P. falciparum*

We genotyped 122 *P. vivax* and 92 *P. falciparum* isolates collected between 2018 and 2021 in the town of Mâncio Lima. Because of the very low parasite densities in most samples –

geometric mean, 19.0 amplicon copies/µL of blood for *P. vivax* (≈0.38 parasites/µL) and 10.5 amplicon copies/µL of blood for *P. falciparum* (≈0.21 parasites/µL) (Table 2, main text) –, only 23.4% of the samples collected during the study waves that were TaqMan-positive for *P. vivax* (including 54 mixed-species infections) and 58.2% of those positive for *P. falciparum* (also including the 54 mixed-species infections) were successfully genotyped for all single-copy microsatellite loci. We additionally genotyped 142 *P. vivax* and 70 *P. falciparum* isolates from surrounding rural areas (Appendix Figure 2).

We found 260 unique haplotypes among 264 *P. vivax* isolates (4 haplotypes shared by 2 isolates each) and 137 unique haplotypes among 162 *P. falciparum* isolates (14 haplotypes shared by 2 to 7 isolates each). The diversity of *P. vivax* populations circulating in Mâncio Lima (mean H_E across six microsatellites of 0.856) remained high throughout the study period (Appendix Table 3), similar to other Amazonian populations characterized with the same microsatellite markers (H_E between 0.66 and 0.83) (*23,26,27*). Interestingly, *P. falciparum* populations from Mâncio Lima were more diverse (mean H_E across six microsatellites of 0.8213) than those from other urban and farming settlements across the Amazon (H_E ranging between 0.39 and 0.52) (*28–31*). A significant LD ($I_A^S > 0$) was observed in parasite populations of both species ($I_A^S = 0.098$, $p < 0.001$, for *P. vivax*; $I_A^S = 0.111$, $p < 0.0001$, for *P. falciparum*), consistent with the local circulation of near-clonal parasite lineages in the region.

Source-Sink Dynamics of Malaria Parasites

"Urban malaria" is often defined as a laboratory-confirmed infection diagnosed in an urban dweller, but a sizeable proportion of these infections may have been imported from the rural surroundings. Individual cases are routinely classified as locally acquired or imported based on travel histories within the past 15 days (*13*), but information regarding recent mobility is entirely dependent on participants′ reports and are often affected by recall bias (*32*).

Our molecular genotyping results indicate that parasite lineages move between the urban and rural areas. Once introduced into the urban area of Mâncio Lima, malaria parasites can propagate locally and originate outbreaks, since the primary vector, *An. darlingi*, is widely distributed across the town (*33,34*). Importantly, parasites can also move from the town to the surrounding rural villages. Therefore, we argue that the town of Mâncio Lima is both a source

and a sink of parasites – i.e., scenario (b) in Appendix Figure 3 – and highlight its central role in maintaining residual malaria transmission in the Juruá Valley region.

Temporal Clustering of Parasite Haplotypes in Juruá Valley

We found evidence for temporal clustering of parasites circulating in the town of Mâncio Lima (Figures 3 and 4, main text). In short, parasites collected in 2019 tend to cluster together in the minimal spanning trees. Moreover, the clusters of genetically related isolates from Mâncio Lima collected in 2019 comprised predominantly parasites from infections detected by microscopy and associated with clinical manifestations (Appendix Figure 4). Interestingly, we found a similar clustering of patent and symptomatic infections in the analysis of the full dataset, which comprises parasite lineages from urban and rural sites in the Upper Juruá Valley (Appendix Figure 5). These findings might result from a near-clonal expansion, in 2019, of more virulent parasites of both species.

However, the observed temporal clustering of symptomatic infections should not be overinterpreted, as it may result, at least in part, from a sample unbalance. Most symptomatic infections – 56 out of 77 *P. vivax* infections and 54 out of 81 *P. falciparum* infections – genotyped had been sampled in 2019 (Table 1, main text). Among them, isolates from patients attending health facilities across the region are overrepresented: they comprise most – 47 out of 56 *P. vivax* infections and 49 out of 54 *P. falciparum* infections – samples from symptomatic infections in 2019 that were genotyped. There were few participants in the community-wide surveys with clinical symptoms who provided samples for genotyping in 2019: only 2 symptomatic *P. vivax* infections and 2 symptomatic *P. falciparum* infections in the town of Mâncio Lima, 6 symptomatic *P. vivax* infections and 2 symptomatic *P. falciparum* infections in Vila Assis Brasil, and only 1 symptomatic *P. vivax* infection in Azul River.

References

1. Corder RM, Paula GA, Pincelli A, Ferreira MU. Statistical modeling of surveillance data to identify correlates of urban malaria risk: A population-based study in the Amazon Basin. PLoS One. 2019;14:e0220980. PubMed https://doi.org/10.1371/journal.pone.0220980

2. Johansen IC, Rodrigues PT, Tonini J, Vinetz J, Castro MC, Ferreira MU. Cohort profile: the Mâncio Lima cohort study of urban malaria in Amazonian Brazil. BMJ Open. 2021;11:e048073. PubMed https://doi.org/10.1136/bmjopen-2020-048073

3. Wilson ML, Krogstad DJ, Arinaitwe E, Arevalo-Herrera M, Chery L, Ferreira MU, et al. Urban malaria: Understanding its epidemiology, ecology, and transmission across seven diverse ICEMR network sites. Am J Trop Med Hyg. 2015;93(Suppl):110–23. PubMed https://doi.org/10.4269/ajtmh.14-0834

4. Monte-Mór R. Modernities in the jungle: extended urbanization in the Brazilian Amazon. Ph.D. thesis. Los Angeles; University of California; 2004.

5. Castriota R, Tonucci J. Extended urbanization in and from Brazil. Environ Plann D Soc Space. 2018;36:512–28. https://doi.org/10.1177/0263775818775426

6. Johansen IC, Ferreira MU. Unintended consequences of ‘development’ in the Amazon: commercial aquaculture and malaria in Mâncio Lima, Brazil. In: Ioris AAR, editor. Environment and development: challenges, policies and practices. London: Palgrave Macmillan, 2021, p. 387–410.

7. Dal’Asta AP, Lana RM, Amaral S, Codeço CT, Monteiro AMV. The urban gradient in malaria-endemic municipalities in Acre: revisiting the role of locality. Int J Environ Res Public Health. 2018;15:1254. PubMed https://doi.org/10.3390/ijerph15061254

8. Filmer D, Pritchett LH. Estimating wealth effects without expenditure data—or tears: an application to educational enrollments in states of India. Demography. 2001;38:115–32. PubMed

9. da Silva-Nunes M, Ferreira MU. Clinical spectrum of uncomplicated malaria in semi-immune Amazonians: beyond the “symptomatic” vs “asymptomatic” dichotomy. Mem Inst Oswaldo Cruz. 2007;102:341–7. PubMed https://doi.org/10.1590/S0074-02762007005000051

10. World Health Organization. Malaria rapid diagnostic test performance: summary results of WHO product testing of malaria RDTs: round 1–7 (2008–2016). Geneva: World Health Organization; 2017 [cited 2024 Jun 3]. https://iris.who.int/bitstream/handle/10665/255836/9789241512688-eng.pdf

11. Putaporntip C, Buppan P, Jongwutiwes S. Improved performance with saliva and urine as alternative DNA sources for malaria diagnosis by mitochondrial DNA-based PCR assays. Clin Microbiol Infect. 2011;17:1484–91. PubMed https://doi.org/10.1111/j.1469-0691.2011.03507.x

12. Barros LB, Calil PR, Rodrigues PT, Tonini J, Fontoura PS, Sato PM, et al. Clinically silent *Plasmodium vivax* infections in native Amazonians of northwestern Brazil: acquired immunity or low parasite virulence? Mem Inst Oswaldo Cruz. 2022;117:e220175. PubMed https://doi.org/10.1590/0074-02760220175

13. Ministry of Health of Brazil. 2010. Practical guidelines for malaria therapy. Ministry of Health of Brazil, Brasília, Brazil [in Portuguese] [cited 2024 Jul 30]. http://bvsms.saude.gov.br/bvs/publicacoes/guia_pratico_malaria.pdf

14. Itoh M, Negreiros do Valle S, Farias S, Holanda de Souza TM, Rachid Viana GM, Lucchi N, et al. Efficacy of Artemether-Lumefantrine for Uncomplicated *Plasmodium falciparum* Malaria in Cruzeiro do Sul, Brazil, 2016. Am J Trop Med Hyg. 2018;98:88–94. PubMed https://doi.org/10.4269/ajtmh.17-0623

15. Ladeia-Andrade S, Menezes MJ, de Sousa TN, Silvino ACR, de Carvalho JF Jr, Salla LC, et al. Monitoring the efficacy of chloroquine-primaquine therapy for uncomplicated *Plasmodium vivax* malaria in the main transmission hot spot of Brazil. Antimicrob Agents Chemother. 2019;63:e01965–18. PubMed https://doi.org/10.1128/AAC.01965-18

16. Karunaweera ND, Ferreira MU, Hartl DL, Wirth DF. Fourteen polymorphic microsatellite DNA markers for the human malaria parasite *Plasmodium vivax*. Mol Ecol Notes. 2007;7:172–5. https://doi.org/10.1111/j.1471-8286.2006.01534.x

17. Anderson TJ, Su XZ, Bockarie M, Lagog M, Day KP. Twelve microsatellite markers for characterization of *Plasmodium falciparum* from finger-prick blood samples. Parasitology. 1999;119:113–25. PubMed https://doi.org/10.1017/S0031182099004552

18. Orjuela-Sánchez P, Brandi MC, Ferreira MU. Microsatellite analysis of malaria parasites. Methods Mol Biol. 2013;1006:247–58. PubMed https://doi.org/10.1007/978-1-62703-389-3_17

19. Haubold B, Hudson RR. LIAN 3.0: detecting linkage disequilibrium in multilocus data. Bioinformatics. 2000;16:847–8. PubMed https://doi.org/10.1093/bioinformatics/16.9.847

20. Hudson RR. Analytical results concerning linkage disequilibrium in models with genetic transformation and conjugation. J Evol Biol. 1994;7:535–48. https://doi.org/10.1046/j.1420-9101.1994.7050535.x

21. Francisco AP, Bugalho M, Ramirez M, Carriço JA. Global optimal eBURST analysis of multilocus typing data using a graphic matroid approach. BMC Bioinformatics. 2009;10:152. PubMed https://doi.org/10.1186/1471-2105-10-152

22. Nascimento M, Sousa A, Ramirez M, Francisco AP, Carriço JA, Vaz C. PHYLOViZ 2.0: providing scalable data integration and visualization for multiple phylogenetic inference methods. Bioinformatics. 2017;33:128–9. PubMed https://doi.org/10.1093/bioinformatics/btw582

23. Salla LC, Rodrigues PT, Corder RM, Johansen IC, Ladeia-Andrade S, Ferreira MU. Molecular evidence of sustained urban malaria transmission in Amazonian Brazil, 2014–2015. Epidemiol Infect. 2020;148:e47. PubMed https://doi.org/10.1017/S0950268820000515

24. Harris PA, Taylor R, Thielke R, Payne J, Gonzalez N, Conde JG. Research electronic data capture (REDCap)—a metadata-driven methodology and workflow process for providing translational research informatics support. J Biomed Inform. 2009;42:377–81. PubMed https://doi.org/10.1016/j.jbi.2008.08.010

25. Vera-Arias CA, Holzschuh A, Oduma CO, Badu K, Abdul-Hakim M, Yukich J, et al. High-throughput *Plasmodium falciparum hrp2* and *hrp3* gene deletion typing by digital PCR to monitor malaria rapid diagnostic test efficacy. eLife. 2022;11:e72083. PubMed https://doi.org/10.7554/eLife.72083

26. Batista CL, Barbosa S, Da Silva Bastos M, Viana SA, Ferreira MU. Genetic diversity of *Plasmodium vivax* over time and space: a community-based study in rural Amazonia. Parasitology. 2015;142:374–84. PubMed https://doi.org/10.1017/S0031182014001176

27. Fontoura PS, Macedo EG, Calil PR, Corder RM, Rodrigues PT, Tonini J, et al. Changing clinical epidemiology of *Plasmodium vivax* malaria as transmission decreases: population-based prospective panel survey in the Brazilian Amazon. J Infect Dis. 2024;229:947–58. PubMed https://doi.org/10.1093/infdis/jiad456

28. Anderson TJ, Haubold B, Williams JT, Estrada-Franco JG, Richardson L, Mollinedo R, et al. Microsatellite markers reveal a spectrum of population structures in the malaria parasite *Plasmodium falciparum.* Mol Biol Evol. 2000;17:1467–82. PubMed https://doi.org/10.1093/oxfordjournals.molbev.a026247

29. Machado RL, Povoa MM, Calvosa VS, Ferreira MU, Rossit AR, dos Santos EJ, et al. Genetic structure of *Plasmodium falciparum* populations in the Brazilian Amazon region. J Infect Dis. 2004;190:1547–55. PubMed https://doi.org/10.1086/424601

30. Ferreira MU, Karunaweera ND, da Silva-Nunes M, da Silva NS, Wirth DF, Hartl DL. Population structure and transmission dynamics of *Plasmodium vivax* in rural Amazonia. J Infect Dis. 2007;195:1218–26. PubMed https://doi.org/10.1086/512685

31. Orjuela-Sánchez P, Da Silva-Nunes M, Da Silva NS, Scopel KK, Gonçalves RM, Malafronte RS, et al. Population dynamics of genetically diverse *Plasmodium falciparum* lineages: community-based prospective study in rural Amazonia. Parasitology. 2009;136:1097–105. PubMed https://doi.org/10.1017/S0031182009990539

32. Johansen IC, Rodrigues PT, Ferreira MU. Human mobility and urban malaria risk in the main transmission hotspot of Amazonian Brazil. PLoS One. 2020;15:e0242357. PubMed https://doi.org/10.1371/journal.pone.0242357

33. dos Reis IC, Codeço CT, Degener CM, Keppeler EC, Muniz MM, de Oliveira FG, et al. Contribution of fish farming ponds to the production of immature *Anopheles* spp. in a malaria-endemic Amazonian town. Malar J. 2015;14:452. PubMed https://doi.org/10.1186/s12936-015-0947-1

34. Alvarez MVN, Alonso DP, Kadri SM, Rufalco-Moutinho P, Bernardes IAF, de Mello ACF, et al. *Nyssorhynchus darlingi* genome-wide studies related to microgeographic dispersion and blood-seeking behavior. Parasit Vectors. 2022;15:106. PubMed https://doi.org/10.1186/s13071-022-05219-5

Appendix Table 1. Expected heterozygosity (H_E) measured for 6 microsatellite markers of *Plasmodium vivax* and *P. falciparum* according to year of sample collection in the urban population of Mâncio Lima, Brazil (2018–2021)

Species	Marker	2018<br>H_E	2019<br>H_E	2020–2021<br>H_E
Plasmodium vivax	MS2	0.702	0.834	0.973
	MS5	0.866	0.919	0.889
	MS6	0.575	0.857	0.776
	MS7	0.798	0.773	0.656
	MS9	0.902	0.767	0.954
	MS15	0.725	0.840	0.936
Average H_E (standard error)		0.761 (0.049)	0.832 (0.023)	0.864 (0.051)
Number of samples tested		45	41	36
Plasmodium falciparum	*Polyα*	0.840	0.674	0.869
	TA60	0.895	0.858	0.784
	TA87	0.735	0.653	0.786
	TA109	0.799	0.621	0.384
	TAA42	0.823	0.684	0.680
	TAA81	0.808	0.595	0.667
Average H_E (standard error)		0.817 (0.021)	0.681 (0.038)	0.695 (0.070)
Number of samples tested		42	20	30

Appendix Table 2. Number of malarial infections diagnosed by microscopy and genus-specific real-time PCR followed by species-specific TaqMan assays, according to the presence or absence of malaria-related symptoms, during 7 consecutive study waves in the urban population of Mâncio Lima, Brazil (2018–2021)*

		Study wave number and diagnostic method															
		1		2		3		4		5		6		7		All waves	
Symptoms	Species	M	PCR	M	PCR	M	PCR	M	PCR	M	PCR	M	PCR	M	PCR	M	PCR
Yes	*Pv*	1	14	8	28	4	15	0	3	2	4	0	1	0	2	15	67
	Pf	2	5	0	7	3	2	1	3	0	1	0	0	0	4	6	25
	Mixed	0	0	0	6	0	2	0	0	0	0	0	0	0	0	0	8
Number tested		204	206	289	289	173	173	195	195	131	131	74	74	49	49	1115	1117
No	*Pv*	12	68	5	87	5	82	3	70	1	40	1	22	0	31	27	400
	Pf	5	17	3	20	2	19	1	15	1	2	0	4	0	2	12	79
	Mixed	0	5	0	9	1	13	0	5	0	9	0	4	0	1	1	46
Number tested		880	887	1416	1416	1402	1402	1573	1574	1544	1544	1794	1796	1993	1994	10603	10613
Total	*Pv*	13	82	13	115	9	97	3	73	3	44	1	23	0	33	42	467
	Pf	7	22	0	27	5	24	2	18	1	3	0	4	0	6	18	104
	Mixed	0	5	0	15	1	15	0	5	0	9	0	4	0	1	1	54
Prevalence of infection, either symptomatic or not, by study wave (%)																	
	Pv	1.2	7.5	0.8	6.7	0.6	6.2	0.2	4.1	0.2	2.6	0.05	1.2	0.0	1.6	0.4	4.0
	Pf	0.6	2.0	0.2	1.6	0.3	1.5	0.1	1.0	0.06	0.2	0.0	0.2	0.0	0.3	0.15	0.9
	Mixed	0.0	0.5	0.0	0.9	0.06	0.9	0.0	0.3	0.0	0.5	0.0	0.2	0.0	0.05	0.01	0.5
Prevalence of malaria by study wave (%)																	
	Pv	0.09	1.3	0.5	1.6	0.2	0.9	0.0	0.2	0.1	0.2	0.0	0.06	0.0	0.1	0.1	0.6
	Pf	0.2	0.5	0.0	0.4	0.2	0.1	0.06	0.2	0.0	0.06	0.0	0.0	0.0	0.2	0.05	0.2
	Mixed	0.0	0.0	0.0	0.3	0.0	0.1	0.00	0.0	0.0	0.0	0.0	0.0	0.0	0.0	0.0	0.07
Number tested		1084	1093	1705	1705	1575	1575	1768	1769	1675	1675	1868	1870	2042	2043	11717	11730
Geometric mean parasite density (95% confidence interval) by study wave and species, Taqman amplicon copies/μL																	
	Pv	18.8 (11.8-29.8)		17.2 (11.1-26.7)		26.1 (14.8-46.1)		15.5 (9.3-25.7)		14.3 (7.2-28.1)		14.8 (6.2-35.7)		31.7 (14.9-67.7)		19.0 (15.2-23.5)	
Number of positives		85		129		108		78		54		27		34		515	
	Pf	22.0 (7.6-63.2)		5.1 (2.5-10.2)		11.1 (3.7-33.5)		10.3 (3.5-30.4)		86.3 (11.8-629.8)		3.2 (1.3-7.9)		5.9 (1.0-33.1)		10.5 (6.8-16.1)	
Number of positives		26		42		35		23		11		8		7		152	

*Dates of study waves were April–May 2018 (wave 1), September–October 2018 (wave 2), May–June 2019 (wave 3), September–October 2019 (wave 4), October–November 2020 (wave 5), April–May 2021 (wave 6), and October–November 2021 (wave 7) (see Figure 2). M = microscopy; PCR = polymerase chain reaction followed by species-specific TaqMan assays. Species: *Pv* = *Plasmodium vivax*. *Pf* = *Plasmodium falciparum*. Prevalence of infection = prevalence of TaqMan-confirmed or microscopy-confirmed infection, regardless of any symptoms. Prevalence of malaria = prevalence of TaqMan-confirmed or microscopy-confirmed infection in the presence of symptoms (reported fever, chills, or headache) within the past 7 days. Numbers of samples with parasite densities estimated by TaqMan ("Number of positives") include mixed-species infections (each species was considered separately to calculate geometric means); however, due to technical issues parasite densities could not be accurately estimated for 6 of 521 *P. vivax*-positive samples and 6 of 158 *P. falciparum*-positive samples. Geometric mean parasite densities for the 54 TaqMan-diagnosed mixed-species infections were 16.1 (95% confidence interval [CI], 8.0-32.3) and 7.1 (95% CI, 3.5- 16.2) amplicon copies/μL for *P. vivax* and *P. falciparum,* respectively.

Appendix Table 3. Comparison of malarial infection diagnosis by microscopy and molecular methods (genus-specific PCR followed by species-specific TaqMan) on 11,717 samples collected during seven study waves in the urban area of Mâncio Lima, Brazil (2018–2021)

	Molecular diagnosis (PCR followed by TaqMan)				
Microscopy	Negative	*P. vivax*	*P. falciparum*	Both species	Total
Negative	11,088	425	92	51	11,656
P. vivax	4	35	0	3	42
P. falciparum	4	2	12	0	18
Both species	0	1	0	0	1
Total	11,096	463	104	54	11,717

Appendix Table 4. Comparison of malarial infection diagnosis by the QuickProfile Malaria Pf/Pv Antigen rapid diagnostic test (RDT) and molecular methods (genus-specific PCR followed by species-specific TaqMan) on 3,343 samples collected during study waves 3 and 4 in the urban area of Mâncio Lima, Brazil (2019)

	Molecular diagnosis (PCR followed by TaqMan)				
RDT	Negative	*P. vivax*	*P. falciparum*	Both species	Total
Negative	3,105	163	46	18	3,317
P. vivax	1	6	0	0	7
P. falciparum	6	0	0	1	7
Both species	0	1	0	1	2
Total	3,112	169	46	20	3,343

Appendix Table 5. Comparison of malarial infection diagnosis by the QuickProfile Malaria Pf/Pv Antigen rapid diagnostic test (RDT) and microscopy on 3,346 samples collected during study waves 3 and 4 in the urban area of Mâncio Lima, Brazil (2019)

	Microscopy				
RDT	Negative	*P. vivax*	*P. falciparum*	Both species	Total
Negative	3,317	6	7	0	3,330
P. vivax	2	5	0	0	7
P. falciparum	6	0	1	0	7
Both species	0	1	0	1	2
Total	3,325	12	8	1	3,346

Appendix Table 6. Factors associated with *Plasmodium vivax* infection, *P. falciparum* infection, and clinical malaria in the urban population of Mâncio Lima, Brazil (data from seven study waves between 2018 and 2021) in unadjusted multiple logistic regression analysis (empty model)*

	Outcome											
	Plasmodium vivax infection				*Plasmodium falciparum* infection				Clinical malaria			
Variables	n/N	OR	95% CI	*P*	n/N	OR	95% CI	*P*	n/N	OR	95% CI	*P*
**Individual-level variables**												
**Age**												
<10	38/1983	1	Reference		13/1938	1	Reference		6/1938	1	Reference	
10–19	156/2748	3.38	1.97–5.80	<0.0001	40/2748	2.27	0.91–5.67	0.080	24/2748	3.02	0.92–9.91	0.067
20–29	97/1731	3.80	2.09–6.91	<0.0001	31/1731	3.12	1.25–7.78	0.015	21/1731	5.08	1.54–16.82	0.008
30–39	97/1976	3.10	1.79–5.37	<0.0001	22/1976	1.75	0.71–4.33	0.224	15/1976	2.83	0.90–8.88	0.074
40–49	58/1309	3.09	1.66–5.78	<0.0001	22/1309	2.79	1.05–7.35	0.038	15/1309	4.62	1.54–13.83	0.006
50–59	34/843	2.23	1.13–4.39	0.021	11/843	2.27	0.78–6.60	0.132	6/843	2.80	0.71–11.01	0.141
60+	49/1177	3.12	1.63–5.98	0.001	19/1177	2.76	1.04–7.28	0.040	13/1177	4.80	1.44–16.00	0.011
		P for trend		0.049		*P* for trend		0.085		*P* for trend		0.012
**Sex**												
Female	265/6160	1	Reference		64/6160	1	Reference		59/6160	1	Reference	
Male	256/5570	1.06	0.80–1.41	0.689	94/5570	1.80	1.25–2.60	0.001	41/5570	0.75	0.51–1.10	0.141
**Any past malaria?**												
No	338/9920	1	Reference		91/9920	1	Reference		49/9920	1	Reference	
Yes	178/1742	1.17	1.05–1.29	0.003	66/1742	1.22	1.11–1.33	<0.0001	51/1742	1.17	1.08–1.27	<0.0001
**Bed net use past night?**												
No	204/4491	1	Reference		59/4491	1	Reference		40/4491	1	Reference	
Yes, not treated	70/1776	0.75	0.51–1.11	0.156	26/1776	0.86	0.48–1.52	0.603	15/1776	0.63	0.34–1.16	0.138
Yes, insecticide-treated	244/5368	0.98	0.72–1.35	0.918	72/5368	1.02	0.65–1.63	0.903	44/5368	0.86	0.52–1.44	0.570
**Household-level variables**												
**Wealth index quartile**												
1 (poorest)	243/4030	1	Reference		79/4030	1	Reference		54/4030	1	Reference	
2	134/2930	0.62	0.45–0.85	0.003	30/2930	0.55	0.32–0.94	0.028	22/2930	0.55	0.31–0.99	0.046
3	84/2605	0.48	0.32–0.73	0.001	31/2605	0.64	0.38–1.10	0.108	18/2605	0.52	0.28–0.96	0.036
4 (wealthiest)	60/2165	0.37	0.23–0.60	<0.0001	18/2165	0.39	0.20–0.75	0.005	6/2165	0.21	0.08–0.52	0.001
		P for trend		<0.0001		*P* for trend		0.006		*P* for trend		<0.0001
**Presence of eave gaps in the house?**												
Yes	437/8825	1	Reference		138/8825	1	Reference		96/8825	1	Reference	
No	84/2894	0.50	0.32–0.77	0.002	20/2894	0.40	0.23–0.72	0.002	4/2894	0.15	0.04–0.35	<0.0001
**Indoor residual spraying (IRS) in the past 12 mo?**												
No	403/9830	1	Reference		122/9830	1	Reference		74/9830	1	Reference	
Yes	104/1697	1.36	0.97–1.90	0.076	34/1697	1.41	0.93–2.14	0.102	25/1697	1.87	1.11–3.14	0.017
**Location of toilet/bathing facilities**												
Inside the house	236/6578	1	Reference		69/6578	1	Reference		41/6578	1	Reference	
Outside the house	276/4808	1.56	1.15–2.11	0.004	86/4808	1.61	1.08–2.39	0.018	58/4808	1.54	0.98–2.42	0.061
Both inside and outside	6/277	0.75	0.30–1.84	0.525	1/277	0.42	0.05–3.79	0.440	1/277	0.55	0.06–4.86	0.590
**Type of bedroom walls**												
Incomplete	239/6490	1	Reference		65/6490	1	Reference		48/6490	1	Reference	
Complete, with gaps	281/5117	1.37	1.01–1.86	0.045	92/5117	1.70	1.13–2.56	0.011	51/5117	1.07	0.67–1.69	0.782
Complete, without gaps	0/39		Not estimated		0/39		Not estimated		0/39		Not estimated	

	Outcome											
	Plasmodium vivax infection				*Plasmodium falciparum* infection				Clinical malaria			
Variables	n/N	OR	95% CI	*P*	n/N	OR	95% CI	*P*	n/N	OR	95% CI	*P*
**Study wave and calendar month and year**												
1 (Apr-May 2018)	87/1093	1	Reference		27/1093	1	Reference		19/1093	1	Reference	
2 (Sep-Oct 2018)	130/1705	0.89	0.60–1.32	0.568	42/1705	1.00	0.60–1.65	0.990	41/1705	1.39	0.75–2.55	0.294
3 (May-Jun 2019)	112/1575	0.80	0.53–1.19	0.264	39/1575	0.98	0.58–1.64	0.941	22/1575	0.76	0.37–1.56	0.457
4 (Sep-Oct2019)	78/1769	0.39	0.26–0.59	<0.0001	23/1769	0.45	0.24–0.85	0.014	6/1769	0.18	0.07–0.48	0.001
5 (Oct-Nov 2020)	53/1675	0.28	0.17–0.44	<0.0001	12/1675	0.25	0.12–0.50	<0.0001	5/1675	0.16	0.06–0.42	<0.0001
6 (Apr-May 2021)	27/1870	0.11	0.07–0.19	<0.0001	8/1870	0.14	0.06–0.31	<0.0001	1/1870	0.03	0.003–0.21	0.001
7 (Oct-Nov 2021)	34/2043	0.12	0.07–0.21	<0.0001	7/2043	0.11	0.04–0.31	<0.0001	6/2043	0.15	0.05–0.43	<0.0001

*n = number of observations with the outcome within the exposure category. N = total number of observations within the exposure category. Numbers of observations may differ for some variables because of missing values. OR = odds ratio. CI = confidence interval. Infection defined as a positive genus-specific PCR result confirmed by species-specific TaqMan assay positive for *Plasmodium vivax* or *Plasmodium falciparum*, regardless of any symptoms. Clinical malaria was defined as a positive genus-specific PCR result confirmed by species-specific TaqMan assay positive for *Plasmodium vivax* or *Plasmodium falciparum* in a study participant who reported symptoms (fever, chills, or headache) within the past 7 d. Note that clinical malaria may be due to *Plasmodium vivax*, *Plasmodium falciparum,* or both species (mixed infections). The total number of participants distributed across exposure categories may vary for some variables because of missing data.

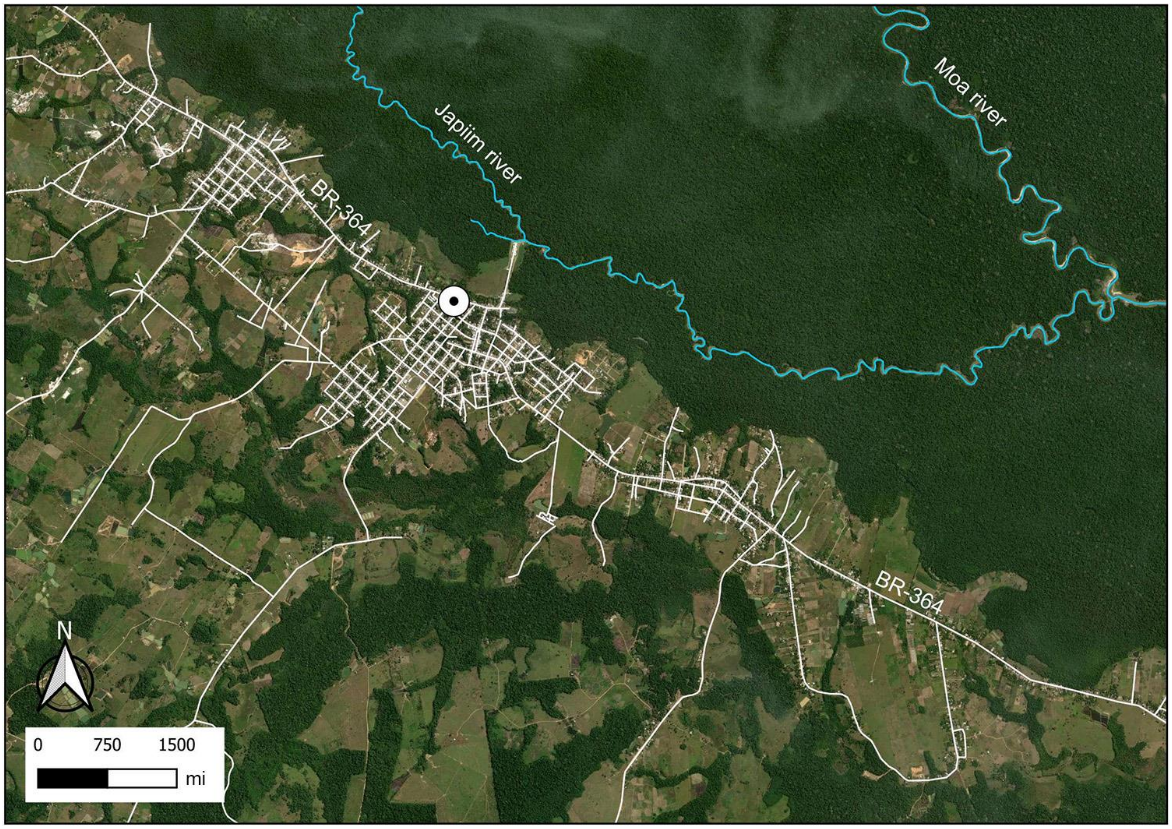

Appendix Figure 1. Urban area of Mâncio Lima, distributed along the main highway (BR-364). The white circle shows the location of city hall, in the center of the town. The northern part of the municipality is covered by rainforest, with some forest fragments remaining in the south. The nearby forest is seasonally flooded, and the town is crisscrossed by small streams. Figure created with QGIS software version 3.8, an open-source Geographic Information System (GIS) licensed under the GNU General Public License (https:// bit.ly/2BSPB2F). Publicly available shape files provided from the Brazilian Institute of Geography and Statistics (IBGE) Web site (https://bit.ly/34gMq0S). Google satellite imagery reproduced observing the conditions for free use (http://bit. ly/2PuYuHt). Adapted from (*6*).

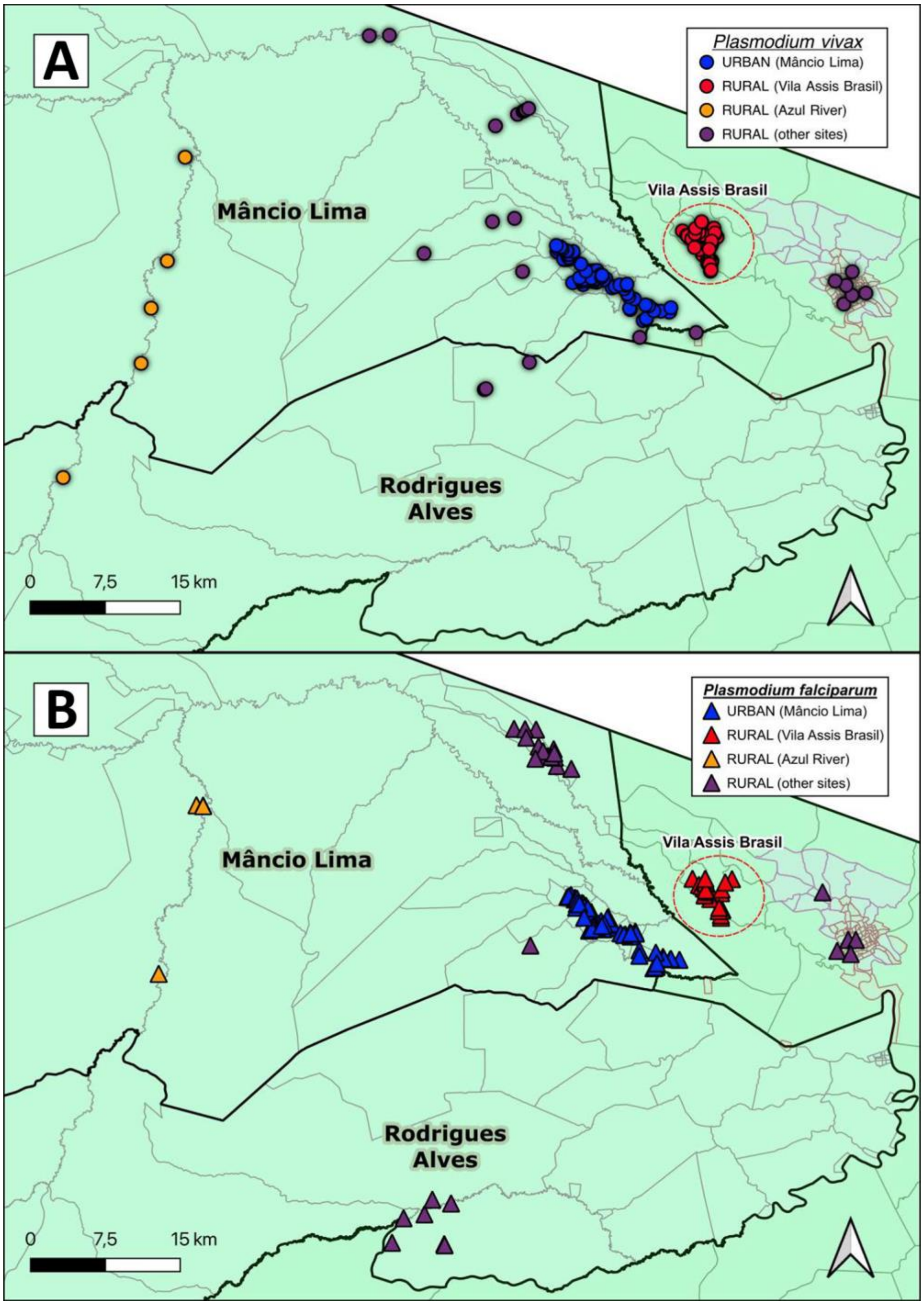

Appendix Figure 2. Geographic origin of genotyped parasites samples from the municipality of Mâncio Lima and the neighboring municipalities of Rodrigues Alves, and Cruzeiro do Sul, upper Juruá Valley, Acre, Brazil. In Panel A, circles indicate the GPS-determined geographic origin of *Plasmodium vivax* samples, either from the urban area of Mâncio Lima (blue) or rural sites (red, orange, or purple). In Panel B, triangles indicate the origin of *P. falciparum* samples from the urban area of Mâncio Lima (blue) or rural sites (red, orange, or purple). Samples with missing GPS location are omitted.

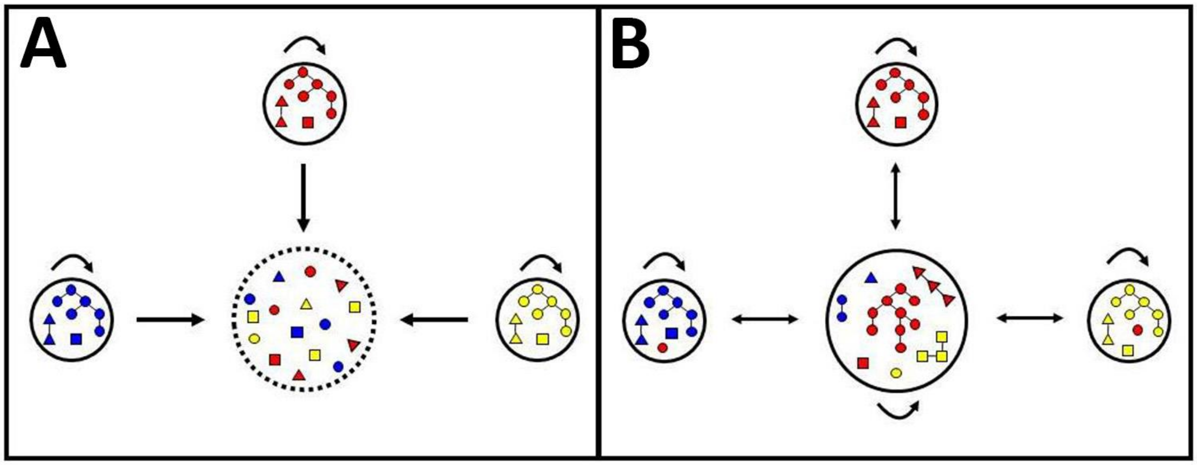

Appendix Figure 3. Hypothetical source-sink relationships between malaria parasites circulating in the town of Mâncio Lima, Acre, Brazil, and the surrounding rural sites: (a) parasites found in the town (colored shapes within the central circle with interrupted contour) are exclusively imported from rural villages (small circles in the periphery) and the town is merely a sink; and (b) parasites found the town (colored shapes within the central circle with continuous contour) may be either imported or locally acquired and can further spread to rural villages (small circles in the periphery) and the town is both a source and a sink of parasites. See the text for further details. Figure reproduced from (*23*).

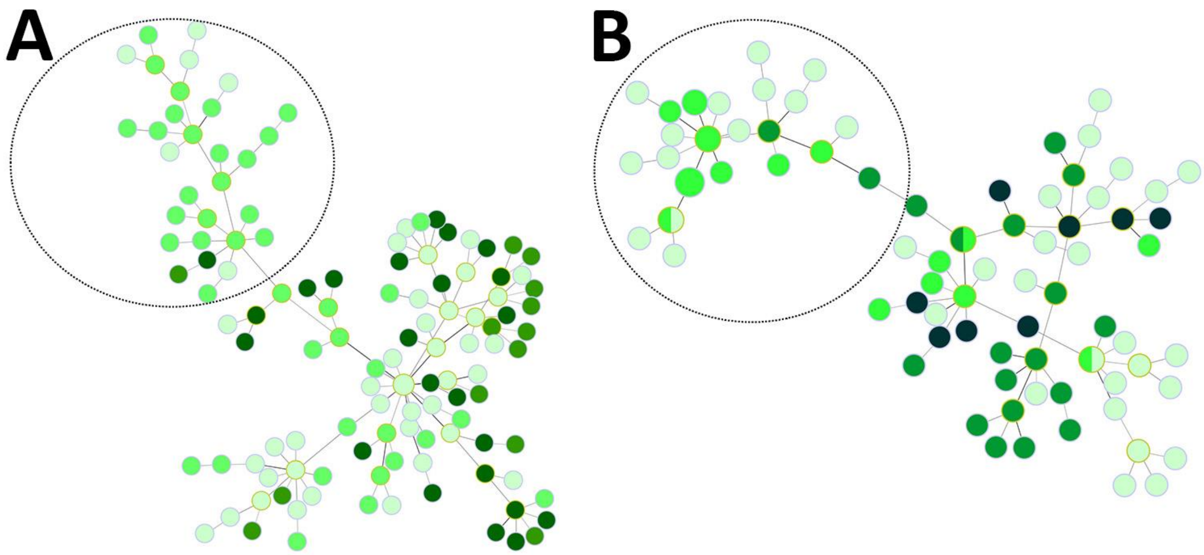

Appendix Figure 4. Minimal spanning trees representing the connectivity among haplotypes found in the parasite populations from the town of Mâncio Lima. Panel A, *Plasmodium vivax* (n = 122 isolates collected between 2018 and 2021); panel B, *P. falciparum* (n = 90 isolates collected between 2018 and 2021). Colored circles represent haplotypes with size logarithmically proportional to the number of isolates sharing them. Lines connect pairs of haplotypes with up to five allele mismatches and the overall network represents the most likely haplotype genealogy generated by the goeBURST algorithm. Haplotypes are color-coded according to the date of sample collection, either 2018 (lighter green), 2019 (light green), 2020 (green), or 2021 (dark green). Clusters of genetically related parasites in the upper part of the tree (encircled) comprise 22 of 62 *P. vivax* samples collected in 2019 (Panel A) and 13 of 20 *P. falciparum* samples collected in 2019 (Panel B), during either study waves or visits to health facilities.

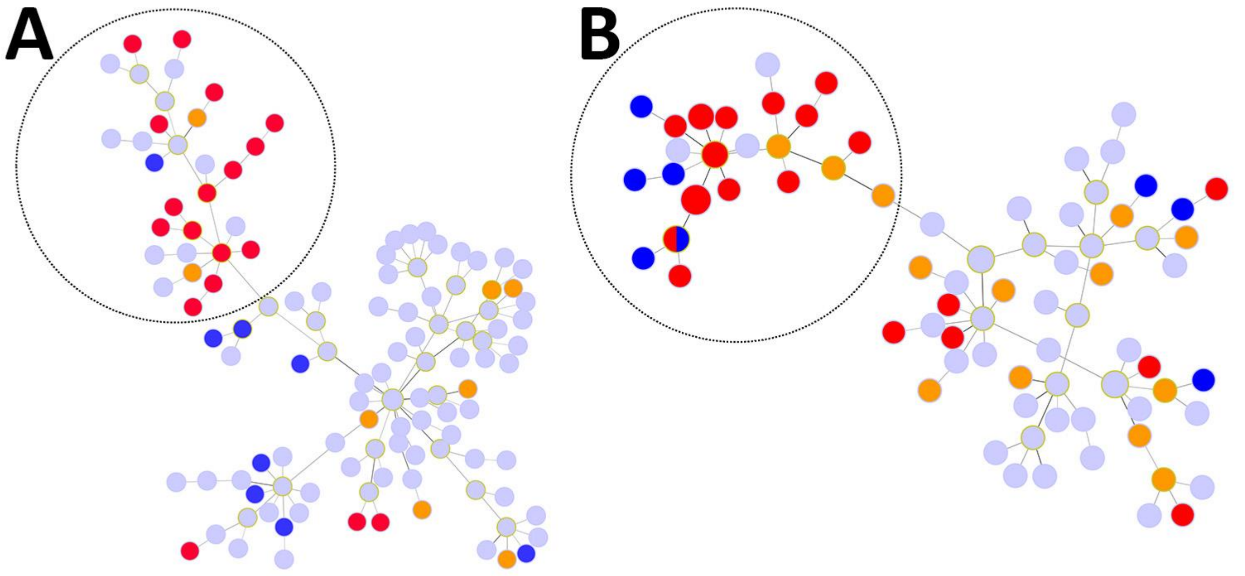

Appendix Figure 5. Minimal spanning trees representing the connectivity among haplotypes found in the parasite populations from the town of Mâncio Lima. Panel A, *Plasmodium vivax* (n = 122 isolates collected between 2018 and 2021); Panel B, *P. falciparum* (n = 90 isolates collected between 2018 and 2021). Colored circles represent haplotypes with size logarithmically proportional to the number of isolates sharing them. Lines connect pairs of haplotypes with up to five allele mismatches and the overall network represents the most likely haplotype genealogy generated by the goeBURST algorithm. Haplotypes are color-coded from the according to the presence of symptoms (red or orange for symptomatic infections; light blue or blue for asymptomatic infections) and microscopy results (orange or light blue for submicroscopic infections and red or blue for patent infections). The clusters of genetically related parasites in the upper left part of the tree (encircled) comprise mostly samples collected from symptomatic and patent infections (red circles).

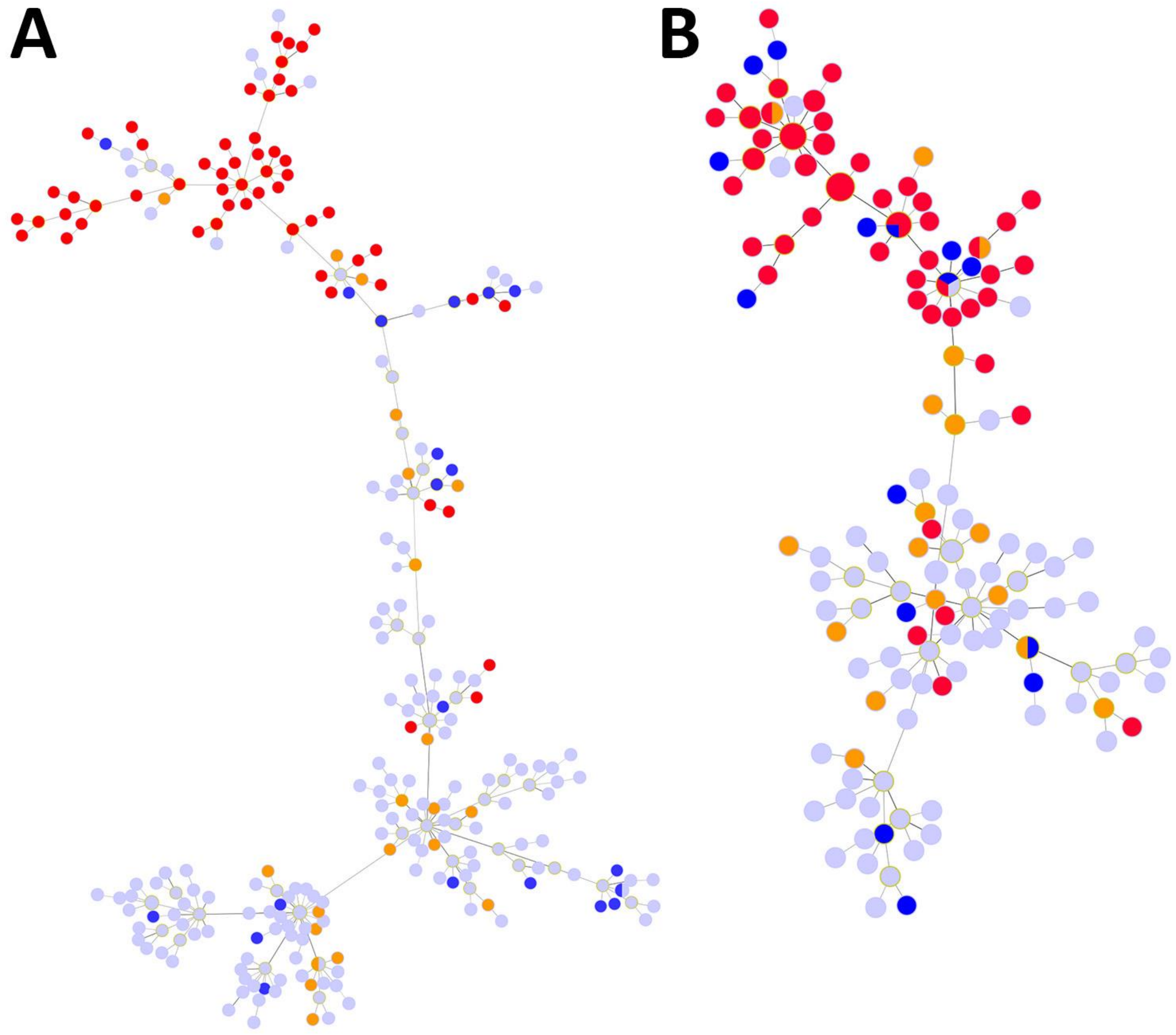

Appendix Figure 6. Minimal spanning trees representing the connectivity among haplotypes found in the parasite populations from the Juruá Valley region. Panel A, *Plasmodium vivax* (n = 264 isolates collected between 2016 and 2021); Panel B, *P. falciparum* (n = 162 isolates collected between 2018 and 2021). Colored circles represent haplotypes with size logarithmically proportional to the number of isolates sharing them. Lines connect pairs of haplotypes with up to five allele mismatches and the overall network represents the most likely haplotype genealogy generated by the goeBURST algorithm. As in Appendix Figure 5, haplotypes are color-coded from the according to the presence of symptoms (red or orange for symptomatic infections; light blue or blue for asymptomatic infections) and microscopy results (orange or light blue for submicroscopic infections and red or blue for patent infections). Parasites collected from symptomatic and patent infections (red circles) are clearly clustered in the upper part of the trees.
